# Rare Variant Analysis of Human and Rodent Obesity Genes in Individuals with Severe Childhood Obesity

**DOI:** 10.1038/s41598-017-03054-8

**Published:** 2017-06-29

**Authors:** Audrey E. Hendricks, Elena G. Bochukova, Gaëlle Marenne, Julia M. Keogh, Neli Atanassova, Rebecca Bounds, Eleanor Wheeler, Vanisha Mistry, Elana Henning, Antje Körner, Dawn Muddyman, Shane McCarthy, Anke Hinney, Johannes Hebebrand, Robert A. Scott, Claudia Langenberg, Nick J. Wareham, Praveen Surendran, Joanna M. Howson, Adam S. Butterworth, John Danesh, Børge G Nordestgaard, Sune F Nielsen, Shoaib Afzal, Sofia Papadia, Sofie Ashford, Sumedha Garg, Glenn L. Millhauser, Rafael I. Palomino, Alexandra Kwasniewska, Ioanna Tachmazidou, Stephen O’Rahilly, Eleftheria Zeggini, Inês Barroso, I. Sadaf Farooqi, Understanding Society Scientific Group, Michaela Benzeval, Jonathan Burton, Nicholas Buck, Annette Jäckle, Meena Kumari, Heather Laurie, Peter Lynn, Stephen Pudney, Birgitta Rabe, Dieter Wolke, EPIC-CVD Consortium, Kim Overvad, Anne Tjønneland, Francoise Clavel-Chapelon, Rudolf Kaaks, Heiner Boeing, Antonia Trichopoulou, Pietro Ferrari, Domenico Palli, Vittorio Krogha, Salvatore Panico, Rosario Tuminoa, Giuseppe Matullo, Jolanda Boer, Yvonne van. der. Schouw, Elisabete Weiderpass, J. Ramon Quiros, María-José Sánchez, Carmen Navarro, Conchi Moreno-Iribas, Larraitz Arriola, Olle Melander, Patrik Wennberg, Timothy J. Key, Elio Riboli, UK10K Consortium, Saeed Al Turki, Carl A. Anderson, Richard Anney, Dinu Antony, María Soler Artigas, Muhammad Ayub, Senduran Bala, Jeffrey C. Barrett, Phil Beales, Jamie Bentham, Shoumo Bhattacharyaa, Ewan Birney, Douglas Blackwooda, Martin Bobrow, Patrick F. Bolton, Chris Boustred, Gerome Breen, Mattia Calissanoa, Keren Carss, Ruth Charlton, Krishna Chatterjee, Lu Chen, Antonio Ciampia, Sebahattin Cirak, Peter Clapham, Gail Clement, Guy Coates, Massimiliano Coccaa, David A. Collier, Catherine Cosgrove, Tony Coxa, Nick Craddock, Lucy Crooks, Sarah Curran, David Curtis, Allan Daly, Petr Danecek, Ian N. M. Day, Aaron Day-Williams, Anna Dominiczak, Thomas Down, Yuanping Du, Ian Dunham, Richard Durbin, Sarah Edkins, Rosemary Ekong, Peter Ellis, David M. Evansa, David R. Fitzpatrick, Paul Flicek, James Floyd, A. Reghan Foley, Christopher S. Franklin, Marta Futema, Louise Gallagher, Tom R. Gaunt, Matthias Geihs, Daniel Geschwind, Celia M. T. Greenwood, Heather Griffin, Detelina Grozeva, Xiaosen Guo, Xueqin Guo, Hugh Gurling, Deborah Hart, Peter Holmans, Bryan Howie, Jie Huang, Liren Huang, Tim Hubbard, Steve E. Humphries, Matthew E. Hurles, Pirro Hysi, Valentina Iotchkova, David K. Jackson, Yalda Jamshidi, Chris Joyce, Konrad J. Karczewski, Jane Kaye, Thomas Keane, John P. Kemp, Karen Kennedy, Alastair Kent, Farrah Khawaja, Margriet van Kogelenberg, Anja Kolb-Kokocinski, Genevieve Lachance, Cordelia Langford, Daniel Lawson, Irene Lee, Monkol Lek, Rui Li, Yingrui Li, Jieqin Liang, Hong Lin, Ryan Liu, Jouko Lönnqvist, Luis R. Lopes, Margarida Lopes, Daniel G. MacArthur, Massimo Mangino, Jonathan Marchini, John Maslen, Iain Mathieson, Peter McGuffin, Andrew M. McIntosh, Andrew G. McKechanie, Andrew McQuillin, Yasin Memari, Sarah Metrustry, Nicola Migone, Josine L. Min, Hannah M. Mitchison, Alireza Moayyeri, Andrew Morris, James Morris, Francesco Muntoni, Kate Northstone, Michael C. O’Donovan, Alexandros Onoufriadis, Karim Oualkacha, Michael J. Owen, Aarno Palotie, Kalliope Panoutsopoulou, Victoria Parker, Jeremy R. Parr, Lavinia Paternoster, Tiina Paunio, Felicity Payne, Stewart J. Payne, John R. B. Perry, Olli Pietilainen, Vincent Plagnol, Rebecca C. Pollitt, David J. Porteous, Sue Povey, Michael A. Quail, Lydia Quaye, F. Lucy Raymond, Karola Rehnström, J. Brent Richards, Cheryl K. Ridout, Susan Ring, Graham R. S. Ritchie, Nicola Roberts, Rachel L. Robinson, David B. Savage, Peter Scambler, Stephan Schiffels, Miriam Schmidts, Nadia Schoenmakers, Richard H. Scott, Robert K. Semple, Eva Serra, Sally I. Sharp, Adam Shaw, Hashem A. Shihab, So-Youn Shin, David Skuse, Kerrin S. Small, Carol Smee, Blair H. Smith, George Davey Smith, Nicole Soranzo, Lorraine Southam, Olivera Spasic-Boskovic, Timothy D. Spector, David St Clair, Beate St Pourcain, Jim Stalker, Elizabeth Stevens, Jianping Sun, Gabriela Surdulescu, Jaana Suvisaari, Petros Syrris, Rohan Taylor, Jing Tian, Nicholas J. Timpson, Martin D. Tobin, Ana M. Valdes, Anthony M. Vandersteen, Parthiban Vijayarangakannan, Peter M. Visscher, Louise V. Wain, Klaudia Walter, James T. R. Walters, Guangbiao Wang, Jun Wang, Yu Wang, Kirsten Ward, Tamieka Whyte, Hywel J. Williams, Kathleen A. Williamson, Crispian Wilson, Scott G. Wilson, Kim Wong, ChangJiang Xu, Jian Yang, Feng Zhang, Pingbo Zhang, Hou-Feng Zheng

**Affiliations:** 1 Wellcome Trust Sanger Institute, Cambridge, UK; 2 Department of Mathematical and Statistical Sciences, University of Colorado-Denver, Denver, CO 80204 USA; 3 University of Cambridge Metabolic Research Laboratories and NIHR Cambridge Biomedical Research Centre, Wellcome Trust-MRC Institute of Metabolic Science, Addenbrooke’s Hospital, Cambridge, UK; 4 The Blizard Institute, Barts and The London School of Medicine and Dentistry, Queen Mary University of London, London, UK; 5 Center for Pediatric Research, University Children’s Hospital Leipzig, Leipzig, Germany; 6 IFB Adiposity Diseases Medical Faculty, University of Leipzig, Leipzig, Germany; 7 Department of Child and Adolescent Psychiatry, Psychotherapy, and Psychosomatics, University Hospital Essen and University of Duisburg-Essen, Essen, Germany; 8 MRC Epidemiology Unit, Institute of Metabolic Science, University of Cambridge School of Clinical Medicine, Cambridge, UK; 9 Cardiovascular Epidemiology Unit, Department of Public Health and Primary Care, University of Cambridge, Cambridge, UK; 10 The National Institute for Health Research Blood and Transplant Unit (NIHR BTRU) in Donor Health and Genomics, University of Cambridge, Cambridge, UK; 11 Department of Clinical Biochemistry and The Copenhagen General Population Study, Herlev and Gentofte Hospital, Copenhagen University Hospital, Copenhagen, Denmark; 12 Faculty of Health and Medical Sciences, University of Copenhagen, Copenhagen, Denmark; 13 Department of Chemistry & Biochemistry, University of California Santa Cruz, Santa Cruz, CA 95064 USA; 14 Institute for Social and Economic Research, University of Essex, Colchester, UK; 15 University of Warwick, Warwick, UK; 16 Department of Pathology, King Abdulaziz Medical City, P.O. Box 22490, Riyadh, 11426 Saudi Arabia; 17 Department of Psychiatry, Trinity Centre for Health Sciences, St James Hospital, James’s Street, Dublin, 8 Ireland; 18 Genetics and Genomic Medicine and Birth Defects Research Centre, UCL Institute of Child Health, London, WC1N 1EH UK; 19 Departments of Health Sciences and Genetics, University of Leicester, Leicester, LE1 7RH UK; 20 Division of Developmental Disabilities, Department of Psychiatry, Queen’s University, Kingston, Ontario, N6C 0A7 Canada; 21 Department of Cardiovascular Medicine and Wellcome Trust Centre for Human Genetics, Roosevelt Drive, Oxford, OX3 7BN UK; 22 European Molecular Biology Laboratory, European Bioinformatics Institute, Wellcome Trust Genome Campus, Hinxton, Cambridge, CB10 1SD UK; 23 Division of Psychiatry, The University of Edinburgh, Royal Edinburgh Hospital, Edinburgh, EH10 5HF UK; 24 Academic Laboratory of Medical Genetics, Box 238, Lv 6 Addenbrooke’s Treatment Centre, Addenbrooke’s Hospital, Cambridge, CB2 0QQ UK; 25 Department of Child Psychiatry, Institute of Psychiatry, Psychology and Neuroscience, King’s College London, 16 De Crespigny Park, London, SE5 8AF UK; 26 NIHR BRC for Mental Health, Institute of Psychiatry, Psychology and Neuroscience and SLaM NHS Trust, King’s College London, London, UK. 16 De Crespigny Park, London, SE5 8AF UK; 27 MRC Social, Genetic and Developmental Psychiatry Centre, Institute of Psychiatry, Psychology and Neuroscience, King’s College London, Denmark Hill, London, SE5 8AF UK; 28 North East Thames Regional Genetics Service, Great Ormond Street Hospital NHS Foundation Trust, London, WC1N 3JH UK; 29 Dubowitz Neuromuscular Centre, UCL Institute of Child Health & Great Ormond Street Hospital, London, WC1N 1EH UK; 30 Leeds Genetics Laboratory, St James University Hospital, Beckett Street, Leeds, LS9 7TF UK; 31 Department of Haematology, University of Cambridge, Long Road, Cambridge, CB2 0PT UK; 32 Department of Epidemiology, Biostatistics and Occupational Health, McGill University, Montreal, Quebec, H3A 1A2 Canada; 33 Institut für Humangenetik, Uniklinik Köln, Kerpener Strasse 34, 50931 Köln, Germany; 34 The Department of Twin Research & Genetic Epidemiology, King’s College London, St Thomas’ Campus, Lambeth Palace Road, London, SE1 7EH UK; 35 Medical Genetics, Institute for Maternal and Child Health IRCCS “Burlo Garofolo”, 34100 Trieste, Italy; 36 Department of Medical, Surgical and Health Sciences, University of Trieste, 34100 Trieste, Italy; 37 Lilly Research Laboratories, Eli Lilly & Co. Ltd., Erl Wood Manor, Sunninghill Road, Windlesham, GU20 6PH UK; 38 MRC Centre for Neuropsychiatric Genetics & Genomics, Institute of Psychological Medicine & Clinical Neurosciences, School of Medicine, Cardiff University, Cardiff, CF24 4HQ UK; 39 Sheffield Diagnostic Genetics Service, Sheffield Childrens’ NHS Foundation Trust, Western Bank, Sheffield, S10 2TH UK; 40 University of Sussex, Brighton, BN1 9RH UK; 41 Sussex Partnership NHS Foundation Trust, Swandean, Arundel Road, Worthing, BN13 3EP UK; 42 University College London (UCL), UCL Genetics Institute, Darwin Building, Gower Street, London, WC1E 6BT UK; 43 Bristol Genetic Epidemiology Laboratories, School of Social and Community Medicine, University of Bristol, Oakfield House, Oakfield Grove, Clifton, Bristol BS8 2BN UK; 44 Computational Biology & Genomics, Biogen Idec, 14 Cambridge Center, Cambridge, Massachusetts 02142 USA; 45 Institute of Cardiovascular and Medical Sciences, University of Glasgow, Wolfson Medical School Building, University Avenue, Glasgow, G12 8QQ UK; 46 Department of Medical and Molecular Genetics, Division of Genetics and Molecular Medicine, King’s College London School of Medicine, Guy’s Hospital, London, SE1 9RT UK; 47 BGI-Shenzhen, Shenzhen, 518083 China; 48 University College London (UCL) Department of Genetics, Evolution & Environment (GEE), Gower Street, London, WC1E 6BT UK; 49 MRC Integrative Epidemiology Unit, School of Social and Community Medicine, University of Bristol, Oakfield House, Oakfield Grove, Clifton, Bristol BS8 2BN UK; 50 University of Queensland Diamantina Institute, Translational Research Institute, Brisbane, Queensland 4102 Australia; 51 MRC Human Genetics Unit, MRC Institute of Genetics and Molecular Medicine, at the University of Edinburgh, Western General Hospital, Edinburgh, EH4 2XU UK; 52 The Genome Centre, John Vane Science Centre, Queen Mary, University of London, Charterhouse Square, London, EC1M 6BQ UK; 53 Cardiovascular Genetics, BHF Laboratories, Rayne Building, Institute of Cardiovascular Sciences, University College London, London, WC1E 6JJ UK; 54 UCLA David Geffen School of Medicine, Los Angeles, California 90095 USA; 55 Lady Davis Institute, Jewish General Hospital, Montreal, Quebec H3T 1E2 Canada; 56 Department of Human Genetics, McGill University, Montreal, Quebec H3A 1B1 Canada; 57 Department of Oncology, McGill University, Montreal, Quebec H2W 1S6 Canada; 58 HeLEX – Centre for Health, Law and Emerging Technologies, Nuffield Department of Population Health, University of Oxford, Old Road Campus, Oxford, OX3 7LF UK; 59 Department of Biology, University of Copenhagen, Ole Maaløes Vej 5, DK-2200 Copenhagen, Denmark; 60 University College London (UCL), Molecular Psychiatry Laboratory, Division of Psychiatry, Gower Street, London, WC1E 6BT UK; 61 Adaptive Biotechnologies Corporation, Seattle, Washington 98102 USA; 62 Human Genetics Research Centre, St George’s University of London, London, SW17 0RE UK; 63 Analytic and Translational Genetics Unit, Massachusetts General Hospital, Boston, Massachusetts 02114 USA; 64 Program in Medical and Population Genetics, Broad Institute of Harvard and MIT, Cambridge, Massachusetts 02142 USA; 65 National Cancer Research Institute, Angel Building, 407 St John Street, London, EC1V 4AD UK; 66 Genetic Alliance UK, 4D Leroy House, 436 Essex Road, London, N1 3QP UK; 67 SW Thames Regional Genetics Lab, St George’s University, Cranmer Terrace, London, SW17 0RE UK; 68 Schools of Mathematics and Social and Community Medicine, University of Bristol, Oakfield House, Oakfield Grove, Clifton, Bristol BS8 2BN UK; 69 Behavioural and Brain Sciences Unit, UCL Institute of Child Health, London, WC1N 1EH UK; 70 Department of Medicine, Jewish General Hospital, McGill University, Montreal, Quebec H3A 1B1 Canada; 71 BGI-Europe, London, EC2M 4YE UK; 72 National Institute for Health and Welfare (THL), Helsinki, FI-00271 Finland; 73 Institute of Cardiovascular Science, University College London, Gower Street, London, WC1E 6BT UK; 74 Cardiovascular Centre of the University of Lisbon, Faculty of Medicine, University of Lisbon, Avenida Professor Egas Moniz, 1649-028 Lisbon, Portugal; 75 Wellcome Trust Centre for Human Genetics, Roosevelt Drive, Oxford, OX3 7BN UK; 76 Illumina Cambridge Ltd, Chesterford Research Park, Cambridge, CB10 1XL UK; 77 National Institute for Health Research (NIHR) Biomedical Research Centre at Guy’s and St Thomas’ Foundation Trust, London, SE1 9RT UK; 78 Department of Statistics, University of Oxford, 1 South Parks Road, Oxford, OX1 3TG UK; 79 Department of Genetics, Harvard Medical School, Boston, Massachusetts 02115 USA; 80 The Patrick Wild Centre, The University of Edinburgh, Edinburgh, EH10 5HF UK; 81 Department of Medical Sciences, University of Torino, 10124 Torino, Italy; 82 Institute of Health Informatics, Farr Institute of Health Informatics Research, University College London (UCL), 222 Euston Road, London, NW1 2DA UK; 83 Usher Institute of Population Health Sciences and Informatics, University of Edinburgh, 9 Little France Road, Edinburgh, EH16 4UX UK; 84 Department of Mathematics, Université de Québec À Montréal, Montréal, Québec H3C 3P8 Canada; 85 Institute for Molecular Medicine Finland (FIMM), University of Helsinki, Helsinki, FI-00014 Finland; 86 Program in Medical and Population Genetics and Genetic Analysis Platform, The Broad Institute of MIT and Harvard, Cambridge, Massachusetts 02132 USA; 87 Institute of Neuroscience, Henry Wellcome Building for Neuroecology, Newcastle University, Framlington Place, Newcastle upon Tyne, NE2 4HH UK; 88 University of Helsinki, Department of Psychiatry, Helsinki, FI-00014 Finland; 89 North West Thames Regional Genetics Service, Kennedy-Galton Centre, Northwick Park Hospital, Watford Road, Harrow, HA1 3UJ UK; 90 University College London (UCL) Genetics Institute (UGI), Gower Street, London, WC1E 6BT UK; 91 Connective Tissue Disorders Service, Sheffield Diagnostic Genetics Service, Sheffield Children’s NHS Foundation Trust, Western Bank, Sheffield, S10 2TH UK; 92 Centre for Genomic and Experimental Medicine, Institute of Genetics and Experimental Medicine, University of Edinburgh, Western General Hospital, Crewe Road, Edinburgh, EH4 2XU UK; 93 Molecular Genetics, Viapath at Guy’s Hospital, London, SE1 9RT UK; 94 ALSPAC & School of Social and Community Medicine, University of Bristol, Oakfield House, Oakfield Grove, Clifton, Bristol BS8 2BN UK; 95 Human Genetics Department, Radboudumc and Radboud Institute for Molecular Life Sciences (RIMLS), Geert Grooteplein 25, Nijmegen, 6525 HP The Netherlands; 96 Department of Clinical Genetics, Great Ormond Street Hospital, London, WC1N 3JH UK; 97 Clinical Genetics, Guy’s & St Thomas’ NHS Foundation Trust, London, SE1 9RT UK; 98 Mackenzie Building, Kirsty Semple Way, Ninewells Hospital and Medical School, Dundee, DD2 4RB UK; 99 Institute of Medical Sciences, University of Aberdeen, Aberdeen, AB25 2ZD UK; 100 School of Oral and Dental Sciences, University of Bristol, Lower Maudlin Street, Bristol, BS1 2LY UK; 101 School of Experimental Psychology, University of Bristol, 12a Priory Road, Bristol, BS8 1TU UK; 102 National Institute for Health Research (NIHR) Leicester Respiratory Biomedical Research Unit, Glenfield Hospital, Leicester, LE3 9QP UK; 103 Maritime Medical Genetics Service, 5850/5980 University Avenue, PO Box 9700, Halifax, Nova Scotia B3K 6R8 Canada; 104 Queensland Brain Institute, University of Queensland, Brisbane, Queensland 4072 Australia; 105 Princess Al Jawhara Albrahim Center of Excellence in the Research of Hereditary Disorders, King Abdulaziz University, P.O. Box 80200, Jeddah, 21589 Saudi Arabia; 106 Macau University of Science and Technology, Avenida Wai long, Taipa, Macau 999078 China; 107 Department of Medicine and State Key Laboratory of Pharmaceutical Biotechnology, University of Hong Kong, 21 Sassoon Road, Pok Fu Lam, Hong Kong; 108 The Centre for Translational Omics – GOSgene, UCL Institute of Child Health, London, WC1N 1EH UK; 109 School of Medicine and Pharmacology, University of Western Australia, Perth, Western Australia 6009 Australia; 110 Department of Endocrinology and Diabetes, Sir Charles Gairdner Hospital, Nedlands, Western Australia 6009 Australia; 111 Department of Public Health, Section for Epidemiology, Aarhus University, Aarhus, Denmark; 112 Department of Cardiology, Aalborg University Hospital, Aalborg, Denmark; 113 Diet, Genes and Environment, Danish Cancer Society Research Center, Copenhagen, Denmark; 114 INSERM, Centre for Research in Epidemiology and Population Health (CESP), U1018, Nutrition, Hormones, and Women’s Health Team, Institut Gustave Roussy, Villejuif, France; 115 Division of Cancer Genetic Epidemiology, German Cancer Research Centre (DKFZ), im Neuenheimer Feld 581, 69121 Heidelberg, Germany; 116 Department of Epidemiology, German Institute of Human Nutrition (DIfE), PotsdamRehbrücke, Germany; 117 WHO Collaborating Center for Nutrition and Health, Unit of Nutritional Epidemiology and Nutrition in Public Health, Department of Hygiene, Epidemiology and Medical Statistics, University of Athens Medical School, Athens, Greece; 118 Hellenic Health Foundation, Athens, Greece; 119 IARC, Lyon, France; 120 Molecular and Nutritional Epidemiology Unit, Centro per lo Studio e la Prevenzione Oncologica-Scientific Institute of Tuscany, Florence, Italy; 121 Epidemiology and Prevention Unit, Fondazione IRCCS Istituto Nazionale dei Tumori, Milan, Italy; 122 Dipartimento di Medicina Clinica e Chirurgia, Federico II University, Naples, Italy; 123 Cancer Registry and Histopathology Unit, Civic- M.P.Arezzo Hospital, ASP Ragusa, Italy; 124 Human Genetics Foundation, Turin, Italy; 125 Department of Medical Sciences, University of Turin, Turin, Italy; 126 Centre for Nutrition, Prevention and Health Services, National Institute for Public Health and the Environment (RIVM), Bilthoven, The Netherlands; 127 Julius Center for Health Sciences and Primary Care, University Medical Center Utrecht, Utrecht, The Netherlands; 128 Department of Community Medicine, Faculty of Health Sciences, University of Tromsø, The Arctic University of Norway, Tromsø, Norway; 129 Department of Research, Cancer Registry of Norway, Institute of Population-Based Cancer Research, Oslo, Norway; 130 Department of Medical Epidemiology and Biostatistics, Karolinska Institutet, Stockholm, Sweden; 131 Genetic Epidemiology Group, Folkhälsan Research Center, Helsinki, Finland; 132 Public Health Directorate, Asturias, Spain; 133 Public Health Institute of Navarra, Pamplona, Spain; 134 Red de Investigación en Servicios de Salud en Enfermedades Crónicas, Madrid, Spain; 135 Epidemiology Department, Murcia Health Authority, Murcia, Spain; 136 Centro de Investigación Biomédica en red en Epidemiología y Salud Pública, Barcelona, Spain; 137 Public Health Division of Gipuzkoa, Instituto Bio-Donostia, Basque Government, CIBERESP, Gipuzkoa, Spain; 138 CRR, Lund University, 20502 Malmö, Sweden; 139 Department of Public Health and Clinical Medicine, Family Medicine, Umeå University, Umeå, Sweden; 140 Cancer Epidemiology Unit, Nuffield Department of Population Health, University of Oxford, Oxford, UK; 141 German Cancer Research Center (DKFZ), Division of Cancer Epidemiology, Heidelberg, Germany

## Abstract

Obesity is a genetically heterogeneous disorder. Using targeted and whole-exome sequencing, we studied 32 human and 87 rodent obesity genes in 2,548 severely obese children and 1,117 controls. We identified 52 variants contributing to obesity in 2% of cases including multiple novel variants in *GNAS*, which were sometimes found with accelerated growth rather than short stature as described previously. Nominally significant associations were found for rare functional variants in *BBS1*, *BBS9*, *GNAS*, *MKKS*, *CLOCK* and *ANGPTL6*. The p.S284X variant in *ANGPTL6* drives the association signal (rs201622589, MAF~0.1%, odds ratio = 10.13, p-value = 0.042) and results in complete loss of secretion in cells. Further analysis including additional case-control studies and population controls (N = 260,642) did not support association of this variant with obesity (odds ratio = 2.34, p-value = 2.59 × 10^−3^), highlighting the challenges of testing rare variant associations and the need for very large sample sizes. Further validation in cohorts with severe obesity and engineering the variants in model organisms will be needed to explore whether human variants in *ANGPTL6* and other genes that lead to obesity when deleted in mice, do contribute to obesity. Such studies may yield druggable targets for weight loss therapies.

## Introduction

Studies focused on severe early onset obesity alone, or obesity with developmental delay and/or dysmorphic features have identified a number of genes harbouring highly penetrant causal mutations^1, 2^. The further characterisation of rare, highly penetrant variants identified in such individuals can provide insights into the cellular and physiological mechanisms involved in energy homeostasis and human obesity, and can identify and/or validate targets for therapeutic intervention. The aim of our study was to examine the prevalence of rare/novel variants in human and mouse obesity genes using high-throughput next-generation sequencing in a large cohort of individuals with severe early onset obesity. Here we describe results from an analysis of 119 candidate genes sequenced in 2,548 individuals with severe, early-onset obesity from the Severe Childhood Onset Obesity Project (SCOOP)^3^ (UK individuals of European ancestry recruited to the Genetics of Obesity Study, GOOS; BMI standard deviation score (SDS) > 3; onset of obesity before the age of 10 years; Methods) from the UK10K project^4^. Our analysis includes 737 SCOOP individuals with whole-exome sequence, and 1,811 additional SCOOP individuals, not consented for whole-exome analysis, in whom we performed targeted sequencing. As this work was performed as part of a consortium (UK10K project), this data was compared to 1,117 individuals with other disorders (e.g. neurodevelopmental and rare disease) in whom exome sequencing and analysis were performed using the same methods. For the purposes of this analysis, these individuals are designated as “controls” (Methods), although we recognise that there are limitations with this design.

## Study Design

SCOOP individuals likely to have congenital leptin deficiency, a treatable cause of severe obesity, were excluded by measurement of serum leptin, and individuals with mutations in the melanocortin 4 receptor gene (*MC4R*) (the most common genetic form of penetrant obesity) were excluded by prior Sanger sequencing.

We focused on six tiers of genes (Methods and Supplementary Table 1): (1) genes known to harbour variants causing human obesity alone (*Obesity Alone*; n = 6); (2) genes in which known variants cause human obesity combined with developmental delay and/or dysmorphology (*Obesity and Delay;* n = 26); (3) genes in which loss-of-function leads to obesity in mice (*LoF Mice*; n = 51); (4) genes in which gain-of-function leads to obesity in mice (*GoF Mice*, n = 5); (5) genes encoding anorectic peptides and their receptors (*Anorectic Molecules*, n = 7), and (6) genes in which loss-of-function is associated with other metabolic phenotypes in mice (*Complex Metabolic Effects*, n = 24). Our aim was to include genes where there was sufficient evidence to indicate disorders with Mendelian inheritance (obesity syndromes) or where complete deletion or overexpression (as opposed to conditional knockouts) causes an obesity phenotype in mice (Methods) (i.e. genes in which inherited loss/gain of function variants might exist). To identify variants more likely to be causally linked to obesity, we focused on ***rare*** (MAF < 1%) and ***novel*** (not seen in the data we used for filtering) variants predicted to be functional (i.e. nonsense variants, missense amino acid substitutions, alterations of conserved splice sites or small insertions/deletions (indels) that introduced a frameshift) (Methods). We filtered our data against approximately 8,000 publicly available sequenced samples, as well as 2,097 exomes (sequenced in parallel on the same platform as our samples) and 3,781 whole-genome sequenced samples also from the UK10K project (Methods).

### Human Obesity Syndrome Genes

**Figure 1:**
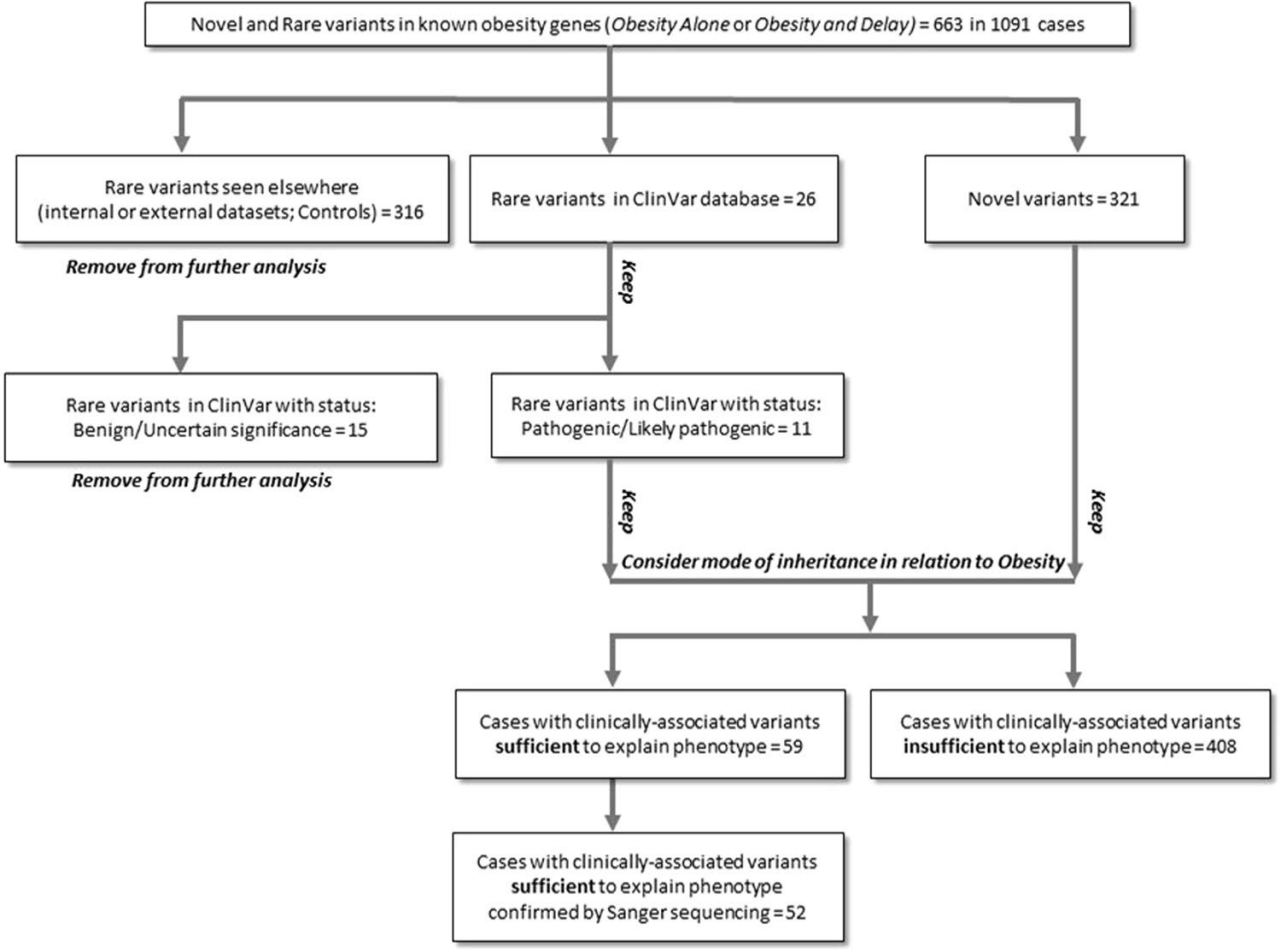
Identification of clinically-associated variants in known human obesity genes. Schematic outlining the analysis strategy.

**Figure 2:**
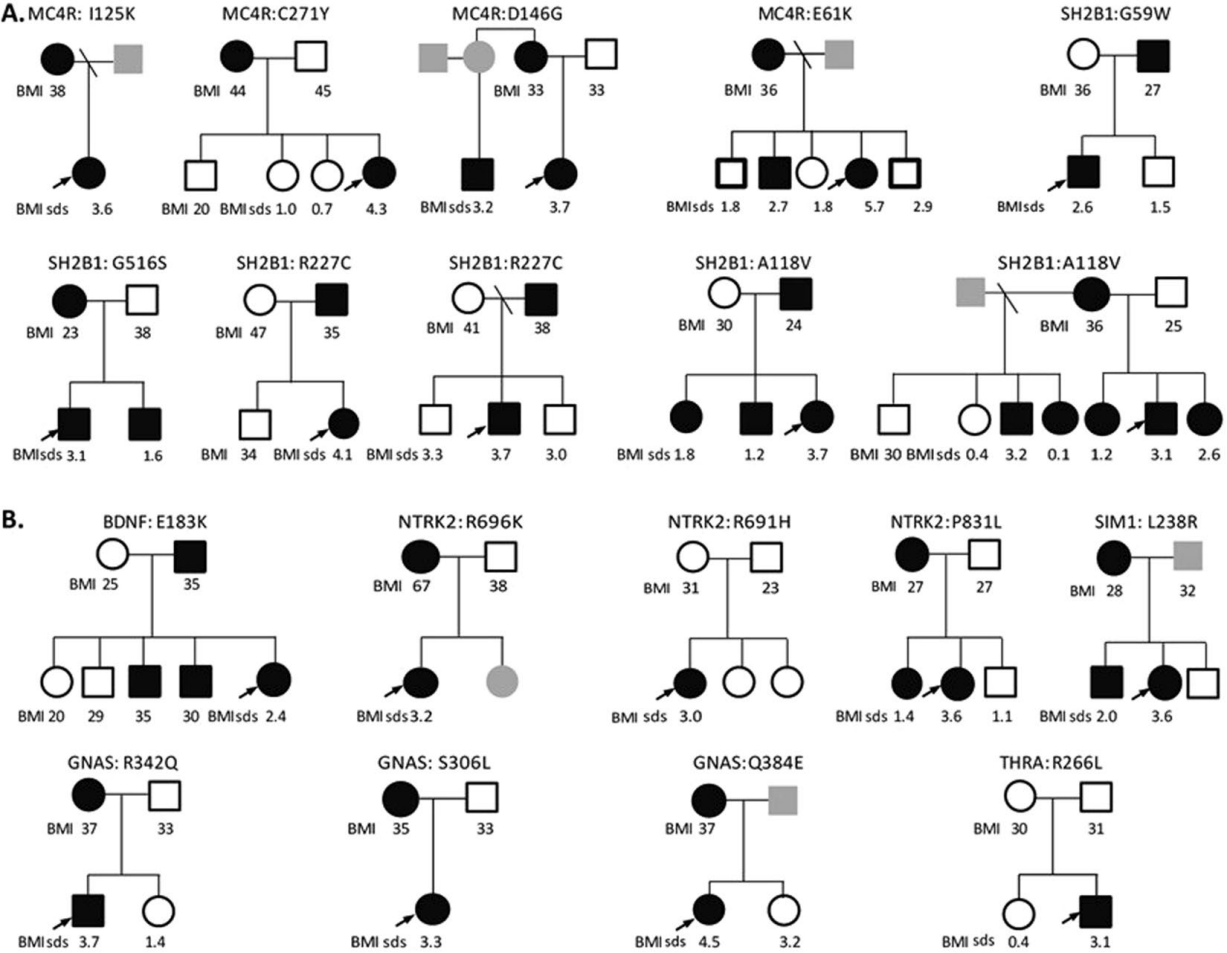
Pedigrees of patients with clinically associated variants contributing to obesity Co-segregation of variants is shown where male (squares) and female (circles) family members consented to genotyping. Heterozygous (filled) and wild-type (empty) mutation carriers are indicated; in some cases, individuals were not available for genotyping (grey). Body mass index (BMI) (>27 kg/m^2^ = overweight;>30 kg/m^2^ = obesity) for adults and BMI standard deviation scores (BMI sds) for children are shown where data was available. (**A)** Obesity alone; (**B)** Obesity and Delay. *MC4R* mutations were excluded by prior Sanger sequencing of all individuals undergoing whole exome sequencing and the majority (1744/1811) of individuals undergoing targeted resequencing. The finding of four *MC4R* mutations in the remaining 82 individuals is in keeping with the prevalence of heterozygous *MC4R* mutations in this cohort as reported previously^5^.

Firstly, we sought to identify rare and novel functional variants in the 32 genes known to cause human obesity, with or without, additional developmental delay and/or dysmorphology features (*Obesity Alone*, or *Obesity and Delay*) (Methods). We identified 11 rare potentially functional variants in ClinVar with pathogenic/likely pathogenic status and 321 novel functional variants, which we confirmed by Sanger sequencing (Fig. 1, Methods). Based on inheritance patterns (where available) and the functional properties of variants that have previously been characterised, variants in these genes may contribute to obesity, sometimes in a non-fully penetrant manner, in 52 (2%) individuals (Supplementary Figure 1 & Supplementary Table 2; Fig. 2, Methods). As variants in *MC4R* account for approximately 5% of severe obesity in this cohort^5^, these findings indicate that > 90% of patients within this cohort do not have their phenotype explained by variants in known human obesity genes. Further analysis exploring the whole-exome in an agnostic manner, with appropriately matched non-disease controls not available in the UK10K project, will be an aim of future investigations.

**Figure 3:**
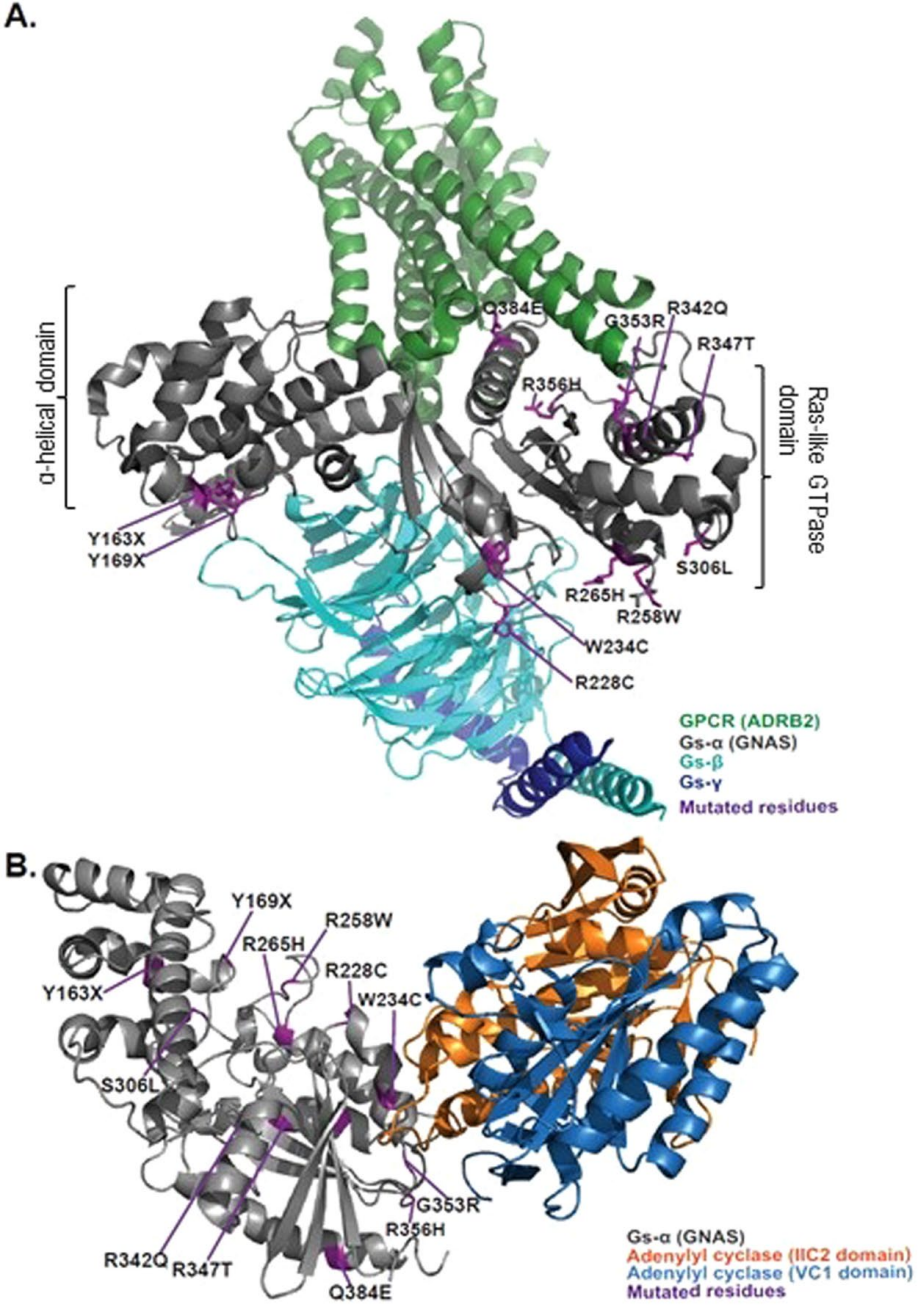
Structural model of variants identified in GNAS. (**A**) Structure of the active state ternary protein complex of G-protein coupled receptor (GPCR) beta 2-adrenergic receptor (ADRB2), and the nucleotide-free Gs heterotrimer, composed of Gs-α(GNAS), Gs-β, and Gs-γ subunits (based on Rasmussen *et al*.^20^, pdb file: 3sn6). The two major domains of GNAS are noted, α-helical domain and Ras-like GTPase domain. The principal interactions between GNAS and GPCRs involve the amino- and carboxy-terminal α-helices. The Ras-like GTPase domain contains most of the catalytic residues necessary for GTP hydrolysis, as well as the Gβγ and effector binding regions (switch regions I, II and III), which change confirmation upon binding to GTP or GDP). (**B**) Structure of the complex of Gs-alpha with the catalytic domains of mammalian adenylyl cyclase (based on Tesmer *et al*.^21^, pdb file: 1azs). Variant residues (purple), and the different components of the complex are highlighted.

Notably, we identified thirteen clinically associated *GNAS* variants in fourteen SCOOP individuals (12 of these confirmed on Sanger sequencing; 0.5%) (Supplementary Table 3). *GNAS* is an imprinted gene in which heterozygous loss-of-function variants are associated with obesity, short stature and skeletal abnormalities, and, when maternally inherited, hormone resistance syndromes^6^. As *GNAS* sequencing has traditionally only been performed in individuals with classical clinical features, our findings suggest that the true prevalence in childhood obesity may be underappreciated. Three of the variants found in our study have been described previously in patients with classical features (p.Y163X^7^; p.R258W^8^, and p.R265H^9^). We identified a novel nonsense variant (p.Y169X) predicted to remove the entire Ras-like GTPase domain and several missense variants predicted to affect downstream signalling by affecting the interaction with G-protein coupled receptors (GPCRs), G-protein β- and γ-subunits, or downstream adenylyl cyclase when mapped onto the protein structure of GNAS (Fig. 3). We confirmed maternal transmission of variants in three out of the five families where parental samples were available for genotyping. Although four *GNAS* variant carriers exhibited endocrinopathies and nine had developmental delay as anticipated, unexpectedly four individuals had accelerated linear growth in childhood (height SDS > 2) rather than short stature (defined as height sds < 2.0) (Supplementary Table 3). Further molecular and physiological studies will be needed to investigate potential genotype-phenotype correlations. As studies in rodents have shown that *Gnas* is imprinted in the paraventricular nucleus of the hypothalamus^10^, the location of the majority of neurons expressing the G-protein coupled receptor MC4R, it is plausible to hypothesize that some *GNAS* variants may contribute to obesity and accelerated linear growth by reducing melanocortin signalling.

Next we formally tested for enrichment of rare, or novel, functional variants in case-control analysis^11^ (Methods). Although no gene attained experiment-wide threshold of p-value < 2 × 10^−4^ (Methods and Supplementary Tables 4–9), four genes (*BBS1*, *BBS9*, *GNAS*, and *MKKS*) known to cause obesity and developmental delay and/or dysmorphology (*Obesity and Delay*) had nominally significant burden test p-values (p-value < 0.05) and a higher burden of variants in cases than controls (Table 1).

**Table 1:** Summary of case-control results.

Gene	Tier	# Variants	Rare alleles in SCOOP TS (N = 1,811)	Rare alleles in SCOOP WES (N = 737)	Rare alleles in Control WES (N = 1,117)	OR		Adjusted OR	p-value SKATO	p-value BURDEN
Rare Functional										
*BBS1*	Obesity and Delay	18	36	18	10	2.368			**0.022**	**0.018**
*BBS9*	Obesity and Delay	20	24	5	5	2.543			0.081	**0.045**
*GNAS*	Obesity and Delay	14	12	4	0	Inf		14.505	**0.015**	**0.008**
*MKKS*	Obesity and Delay	19	90	25	28	1.801			**0.006**	**0.007**
*ANGPTL6*	Mouse LOF	12	18	10	4	3.073			**0.043**	**0.027**
Novel Functional										
*GNAS*	Obesity and Delay	13	11	4	0		Inf	13.629	**0.019**	**0.010**
*CLOCK*	Mouse LOF	9	8	2	0		Inf	9.208	0.066	**0.036**

Summary of case-control results in genes with nominally significant (p-value < 0.05, bold) burden of *Rare* (top) or *Novel* (bottom) functional variants in SCOOP cases (N = 1,811 with targeted sequence and N = 737 with whole-exome sequence), compared to 1,117 controls with whole-exome sequence data from the UK10K project. The number of variants (# variants) per gene are shown, as well as the number of alleles in cases and controls, odds ratios (OR) and p-values from SKAT-O (p-value SKATO) and from burden tests (p-value BURDEN). For variants not detected in controls an adjusted odds ratio (Adjusted OR) was calculated by adding 0.5 to the number of alleles in each cell of the two-by-two table.

### Mouse Obesity Genes

**Figure 4:**
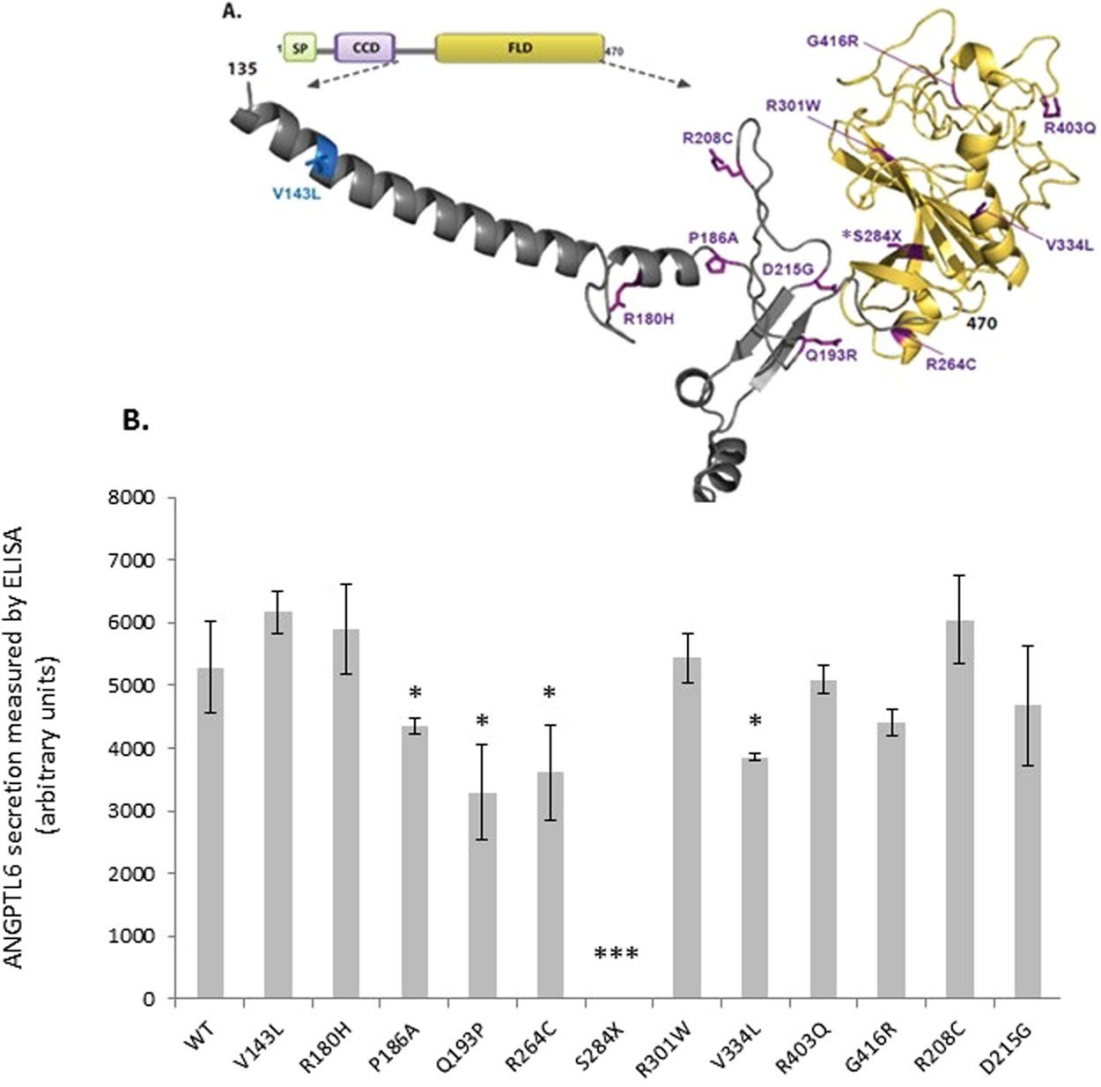
Functional characterisation of ANGPTL6 variants location and functional effect (**A**) Human ANGPTL6 protein and location of the genetic variants. Schematic of the human ANGPTL6 protein with the three recognisable domains: signal peptide (SP) in green, coiled-coil domain (CCD) in purple, and fibrinogen-like domain (FLD) in yellow. As all genetic variants identified are located predominantly in the FLD, the partial tertiary structure of the C-terminal protein portion (amino acids 135–470), based on previously solved fibrinogen structure (1lwu) is presented. The single variant seen in controls only is shown in blue, variants identified in cases are in purple, the S284X variant is starred. (**B**) Functional characterisation of ANGPTL6 variants. Cells were transiently transfected with constructs encoding wild-type (WT) or mutant ANGPTL6; levels of protein secretion into the media were measured by ELISA; means+/− standard deviation (SD) for experiments performed in triplicate are shown (results were confirmed by Western blotting; data not shown). Statistical significance was measured using unpaired T-test with Welch’s correction using the GraphPad Prism software. *p < 0.05; ***p < 0.001.

We found a higher burden of rare variants in cases than controls for two genes known to cause obesity when disrupted in mice - *ANGPTL6* and *CLOCK* (Table 1). Testing the functional consequences of *CLOCK* variants in cells is not straightforward as the molecular mechanisms that lead to obesity are not fully understood. As such, we focused on verifying the accuracy of the computationally predicted function of all twelve rare *ANGPTL6* variants (Methods, Fig. 4, Supplementary Table 10). All variants found in cases (but not the one variant found exclusively in controls, V143L) were predicted to affect the fibrinogen-like domain of the protein (Fig. 4a)*. ANGPTL6* is predominantly expressed in the liver but is also expressed in white adipose tissue and kidney. To evaluate the effect of these variants, HEK293 cells were transiently transfected with constructs encoding wild type and variant forms of *ANGPTL6*. Whereas wild-type ANGPTL6 was readily detected in the medium, several mutants reduced, and the S286X mutant abolished, secretion of *ANGPTL6* (Fig. 4b). Incorporation of this functional information did not qualitatively change the results of the *ANGPTL6* gene-based test (Supplementary Table 10). However, limiting the analysis to the single variant that completely abolished protein secretion (p.S286X, rs201622589) led to an adjusted OR = 10.13 and p-value = 0.028 (Fisher’s p-value = 0.041) suggesting that the association signal at this gene is primarily driven by this variant (Supplementary Table 10). To increase power, and given that there were no additional cases of European descent from our obesity cohort, we analysed existing exome-chip data from an additional 253,587 unrelated European, non-Finnish population controls (Methods, Supplementary Table 11). In an updated analysis with the 2,548 cases and 253,587 controls, we observed an odds ratio (OR) = 2.90 and a Fisher’s p-value = 0.0022, which did not reach experiment or exome-wide significance. Further analysis of 1,436 non-overlapping obesity cases and 1,954 non-overlapping controls from two studies did not lend additional support, and when combined with the original data, yielded an overall OR = 2.34 and chi-squared p-value = 0.0060 (total 3,984 cases vs 256,658 controls, Supplementary Table 11). The results are similar when limiting to non-obese controls (Supplementary Table 12). These findings highlight the difficulty in studying very rare variants in complex diseases, and potentially the effect of winner’s curse, reinforcing the need for extremely large sample sizes^12^. Of interest, mice with targeted deletion of *Angptl6* that survive to birth (20%) develop marked obesity, have increased food intake, reduced energy expenditure, exhibit lipid accumulation in liver and muscle, and develop insulin resistance^13^. Conversely, mice with targeted overexpression of *Angptl6* are lean, insulin sensitive and are protected from diet-induced obesity^13^. As ANGPTL6 is a liver-derived circulating peptide, and thus could potentially be manipulated for therapeutic purposes^14^, further genetic studies in larger cohorts and experimental studies in mice and humans are necessary to explore its role in obesity and potential utility as an anti-obesity drug target.

### Power and Gene Set Analysis

**Figure 5:**
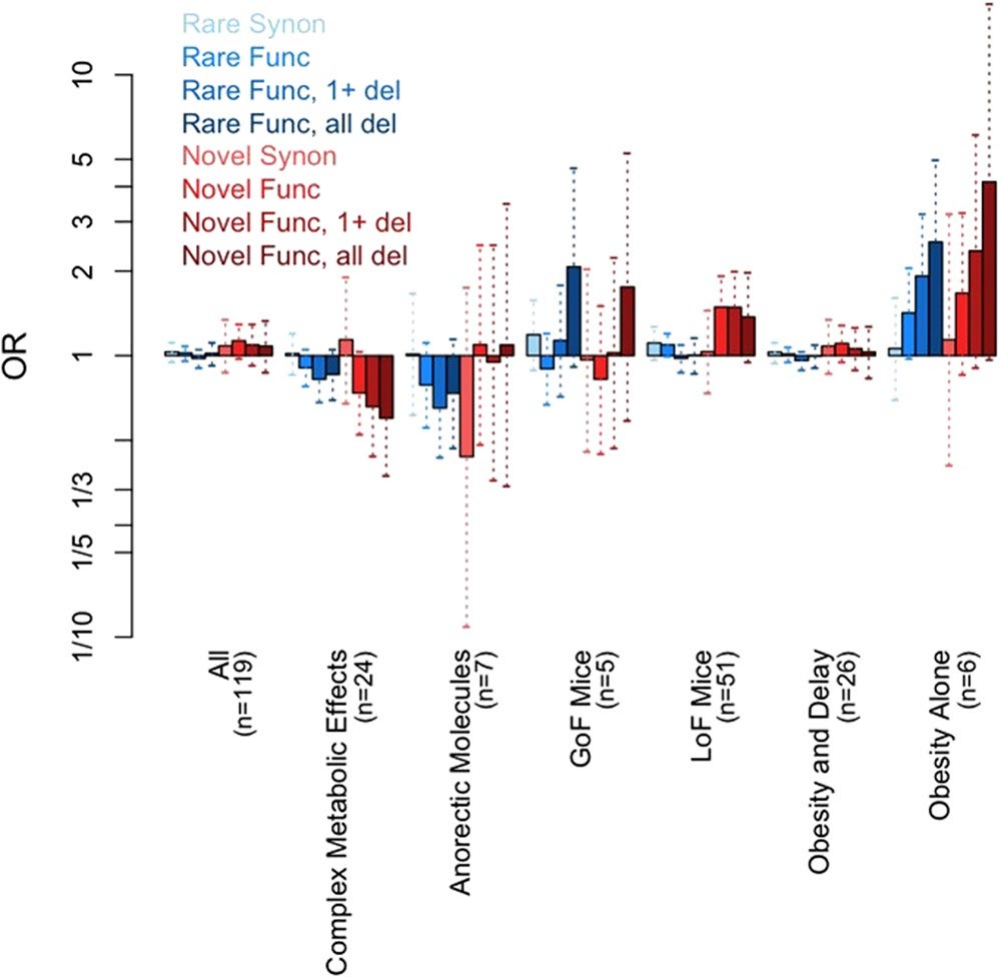
Tiered analysis of obesity candidate genes sets. ORs (bars) and 95% (dotted vertical lines) confidence intervals for each of the six gene tiers and across all 119 genes combined each for eight different filtering scenarios by MAF (*Rare* or *Novel*) and functional prediction (synonymous - *Synon*, functional - *Func*, functional with at least 1 deleterious consequence prediction – *Func*, *1 + del*, and functional with all deleterious consequence predictions - *Func*, *all del*).

Assuming an experiment-wide significance level of 2 × 10^−4^ and that 30% of rare (MAF < 1%) variants within a given gene are causal we have very limited power (~20%) to detect an association to a gene region using our sample of 2,548 cases and 1,117 controls (Supplementary Figure 2, Online Methods, Supplementary Note 4)^12^. As there is evidence that looking across group of genes with stronger priors may increase power^15–17^, we tested for association of rare or novel functional variants within each of the six candidate gene sets. We further filtered variants with respect to their likelihood of being deleterious and performed six nested tests for each gene set (Methods). Although none of the analyses of tiered gene sets reached experiment-wide significance (Supplementary Table 13, Fig. 5), we see an increase in OR as we focus on rarer variants with more evidence of predicted deleterious effect within the *Obesity Alone* gene set. This suggests that focusing on extremely rare variants with strong evidence of being deleterious may be a good strategy for diseases with a complex genetic architecture, which likely includes rare, highly penetrant alleles. Our power analyses also suggests that restricting the MAF threshold to be near the very rare allele frequency of that expected for the causal variants, rather than a more lenient MAF threshold of 1%, increases power (Supplementary Figure 3). Finally, there are nominally significant signals (p-value < 0.05) within the *LoF Mice* candidate gene set for the novel, functional variant tests suggesting that within this rather large gene set (n = 51) there may be additional genes associated with human early onset obesity (Fig. 5).

## Discussion

In summary, analysis of sequence data in 119 genes across 2,548 severely obese children and 1,117 other disease controls from the UK10K project identified variants in known obesity genes, contributing to obesity in 2% of children within this cohort, in whom mutations in MC4R and leptin had been excluded. It also highlighted that *GNAS* mutations may be more prevalent in human obesity than previously thought, and that the spectrum of phenotypic consequences may be broader and more heterogeneous than previously described (6). Additional detailed genotype-phenotype studies will be required to further elucidate the molecular underpinnings of the physiological consequences of these variants.

Taking the information generated in this study together with the published literature, we suggest that molecular genetic investigations, including testing known obesity genes, or agnostic exome-wide or genome-wide approaches (as these become more established at the point of care), should become part of the assessment for a child presenting with severe obesity (BMI SDS > 3) in the absence of other syndromic features. Genetic studies can allow the identification of congenital leptin deficiency which is entirely treatable with recombinant leptin therapy^18^. Also, early reports suggest that patients with mutations that disrupt POMC signalling may be effectively treated with Setmelanotide (a MC4R agonist)^19^. Finally, our data suggest that focusing on very rare, deleterious variants may increase power to find genetic association. Further detailed investigation of genes that lead to obesity when deleted in mice may identify new pathophysiological mechanisms involved in human obesity that can be targeted for drug discovery.

## Methods

### Sample Sets

All studies were approved by the Cambridge Local Research Ethics Committee and all participants and their parents gave written informed consent. All methods were performed in accordance with the relevant laboratory/clinical guidelines and regulations.

The SCOOP cohort^3^ was studied as part of the UK10K consortium^4^. Data was compared to subsets within the neurodevelopmental and rare disease groups that were consented for use as controls. Details and further information about the UK10K project can be found at http://www.uk10k.org/ and in the UK10K consortium paper from 2015^4^.

We gathered the genotypes for the variant S284X (rs201622589) in 202,981 unrelated population controls with European non-Finnish ancestry from existing cohorts (UKHLS^22^, Fenland, EPIC Norfolk^23^, CCHS^24–26^, CGPS^24–26^, CIHDS^24–26^, EPIC-CVD^27^, UK Biobank^28^ and ExAC^29^) and in two additional studies of obese children and healthy or thin controls^30, 31^ including one from the Leipzig Childhood Obesity Cohort^32^. (Supplementary Note 1)

### Sequencing, Variant Calling, and Quality Control

Targeted Sequencing (TS) and Whole Exome Sequencing (WES) was performed as described elsewhere (refs 33 and 4 respectively). All UK10K WES samples from all arms of the exome study available at the time of the variant calling and SCOOP TS samples (N = 5,233 and 2,819 respectively) were called together on the non-redundant targets + /− 100 bp using multi-sample calling. Using SAMtools^34^, a BCF file was created and the site genotype likelihoods were calculated. Variants (SNPs and Indels) were called using BCFtools. Indels were left-aligned using ‘vcf norm’ from the htslib package. Variants were filtered for sequencing and genotype quality at the site and genotype level using *vcf-annotate* ^35^. Additional details in Supplementary Note 2.

### Variant Annotation

All variant annotation was applied using the GRCh37 human reference. Variants were annotated with rsIDs from dbSNP 137, and allele frequencies from the final 1000Genomes Phase 1 integrated (v3) callset^36^, the NHLBI Exome Sequencing Project (ESP) v2^37^, and the UK10K WGS sample set^4^. The Ensembl Variant Effect Predictor (http://www.ensembl.org/info/docs/variation/vep/index.html)^38^ v2.8 with Ensembl 66 was used to add variant consequence annotations including the predicted deleteriousness of each missense variant as predicted by SIFT^39, 40^, PolyPhen^41, 42^, and Condel^43^. For the 32 known human obesity genes, only the variant consequences on the most clinically relevant transcript were considered for further analysis. For the other 87 candidate obesity genes, the most severe consequence on any transcript was retained for each variant (Supplementary Table 1). Using Sequence Ontology terms^44, 45^, variant consequences were defined as functional (i.e. essential splice site, stop gained, stop lost, complex indel, frameshift coding, non synonymous coding, within mature miRNA, partial codon).

To identify ***rare*** variants, we used the thirteen UK10K WES sample sets that were not obese sample sets and were not included as controls in this study (Supplementary Note 1). These sample sets were processed in parallel with our cases and controls and thus provide a similar sequencing coverage and depth across the called regions. We removed variants that had a MAF > 1% across all or a MAF > 10% in any of the thirteen sample sets. We then removed variants with a MAF > 1% in any of seven additional sample sets: 1. UK10K WGS sample set^4^, 2–3. European and African American NHLBI ESP v2 sample sets ^37^, and 4–7, the four continent sample sets (AFR, AMR, ASN, EUR) from 1000 Genomes Phase 1 integrated v3 call set^36^. ***Novel*** variants were identified as sites not seen in any of the internal or external datasets used for MAF annotation, i.e. novel at the time of this study.

### Sample quality control

Samples were identified as contaminated using a combination of two methods: VerifyBamID v1.0^46^ and “fraction skewed hets”^4^. For the TS samples, the FREEMIX value was estimated using 11,250 high quality, autosomal, biallelic SNPs with an alternate AF ≥ 0.01, and a call rate ≥ 0.5 in both the TS sample set and the 1000Genomes Project Phase 1 v3^36^. Of the 2,819 TS SCOOP samples, the 784 WES SCOOP samples, and the 1427 WES samples used as controls 37, one, and nine were excluded due to contamination respectively. Sixteen non-contaminated TS samples were excluded based on a mean sample read-depth 3 SD below the average for all samples (i.e. mean sample read-depth < 12.09). Genotype concordance for 436 WES SCOOP samples and 1,035 TS SCOOP samples for which we had both sequence and GWAS data^3^ was also calculated. Four WES samples and six TS samples with a concordance rate below 90% were identified and excluded. A set of highly polymorphic markers (MAF > 0.3) was genotyped and compared to the sequencing calls from each sample. Non-concordant WES samples were removed prior to variant calling and are not included in the original sequencing numbers. Eighty-one TS samples with low concordance were removed after variant calling. Three TS samples were removed due to having an extremely high genotype missing rate of > 50%.

To identify non-European samples, we calculated principal components (PCs) from the 1000Genomes Phase I integrated call set^36^ using either EIGENSTRAT v4.2^47^ or LASER 2.0^48^ for the WES and TS samples respectively (Supplementary Note 3). Of the 2,676 TS samples, 837 were classified as non-European due to genetic ancestry and three were excluded due to a reported non-European ancestry resulting in 1,836 good quality TS samples of European ancestry. Of the 779 WES SCOOP samples, 37 samples were classified as non-European due to genetic ancestry resulting in 742 good quality WES SCOOP samples of European ancestry. Of the 1,418 control samples, 146 were identified as being non-European leaving 1272 high quality WES controls.

Known and cryptic relatedness was identified by estimating pairwise identity by descent using PLINK v1.07^49^. Genetic relationships within the WES sample set is described in detail elsewhere^4^. We removed individuals sequentially by: (1) largest number of relationships, (2) diseased controls, non-diseased controls, cases, (3) lower mean depth of sequencing. Five SCOOP cases and 155 controls were removed resulting in 737 SCOOP WES cases and 1,117 WES controls all of high quality, unrelated, and European ancestry. A similar process was used to identify genetic relationships within the TS sample set and between the TS and WES sample sets (Supplementary Note 3). This resulted in 25 additional exclusions (18 TS cases related to WES cases and seven related within the TS sample set) for a total of 1,811 high quality, unrelated, TS samples of European ancestry.

### Candidate Gene Sets

We performed database searches using keywords ‘obesity’, ‘growth’, ‘size’, ‘adipose tissue’, (details below) and manually curated the results to arrive at a set of six tiers of candidate gene with definite or likely links to obesity. The Online Mendelian Inheritance of Man (OMIM database, http://www.omim.org/) accessed in February, 2013^50^ was used to identify 32 genes (Supplementary Table 1) which directly lead to human obesity. These were further categorised into two groups based on consistent clinical features: genes characterised by “Obesity alone” (*Obesity Alone*; n = 6), and “Obesity and developmental delay and/or dysmorphology” (*Obesity and Delay;* n = 26). Any putative list of candidate genes has limitations and other genes that contribute to energy homeostasis/obesity in animal models deserve interrogation in the future.

Genes that cause obesity when disrupted in rodents were identified using the Mouse Genome Informatics Database (MGI, http://www.informatics.jax.org/)^51^ and the Rat Genome Database (RGD, http://rgd.mcw.edu)^52^ both accessed in February 2013, as well as published research (www.ncbi.nlm.gov/Pubmed). Eighty-seven genes were associated with a growth/size phenotype when perturbed in rodent animal models. We evaluated the strength of evidence for a role of each gene in obesity phenotype and divided them into four further categories, specifically: Loss of function associated with obesity in mice (*LoF Mice*; n = 51); gain of function associated with obesity in mice (*GoF Mice*, n = 5); anorectic peptides and receptors (*Anorectic Molecules*, n = 7); loss of function associated with other metabolic phenotypes in mice (*Complex Metabolic Effects*, n = 24) (Supplementary Table 1).

### Sequence validation of rare functional variants

Novel, functional variants in all 119 candidate genes were taken forward for validation by conventional Sanger sequencing (90% validation rate). Briefly, customized PCR primers were designed+/−250 bp surrounding the variant; and sequencing was performed using BigDye Terminator v3 kit (Applied BioSystems) and analysed by capillary electrophoresis on an ABI3730 DNA Analyzer platform (Applied Biosystems), according to the manufacturers’ instructions. Familial segregation analysis of variants was performed where family samples were available.

### Identification of clinically-associated variants in known human obesity genes

The strategy for identification of clinically-associated variants in known human obesity genes is outlined in Fig. 1. Briefly, novel and rare variants in the known human obesity genes were filtered using the ClinVar database (http://www.ncbi.nlm.nih.gov/clinvar/). We retained novel variants and those with a ClinVar status of Pathogenic/Likely pathogenic (N = 332).

### Structural Analysis of GNAS Variants

For structural analysis of the genetic variants and prediction of their impact on protein function, we modelled their location in relation to previously solved protein crystal structures of GNAS in complex with either G-protein coupled receptor (ADRB2) or downstream effector (adenylyl cyclase). Specifically, the first model represented in Fig. 3a is based on crystal structure of the active state ternary protein complex of GPCR beta-2-adrenergic receptor (ADRB2), and the nucleotide-free Gs heterotrimer, composed of Gs-α(GNAS), Gs-β, and Gs-γ subunits (ref. 20, PDB number: 3SN6, www.rcsb.org). The second model, represented in Fig. 3b, is based on complex of GNAS with the catalytic domains of mammalian adenylyl cyclase (ref. 21, PDB number: 1AZS, www.rcsb.org). The structural representations using ribbon-depicted models were generated using the Open-Source PyMOL Molecular Graphics System, Version 1.7.x Schrödinger, LLC (http://pymol.org).

### Association Analyses

We implemented the optimal Sequence Kernel Association Test SKAT-O^11, 53^ with the SKAT R package v1.1.2^54^ using options *method* = ”optimal.adj” for SKAT-O and *r.corr* = *1* for burden. For each gene and candidate gene set, we performed two primary tests using the burden test: 1. rare functional (*Rare Func*) or (2) novel functional (*Novel Func*). We performed secondary tests restricting either to variants that were predicted to be deleterious by at least one of the three algorithms (i.e. SIFT, PolyPhen, and Condel) (*1* + *del*) or to variants that were predicted to be deleterious by all three algorithms (*all del*). For SIFT and Condel, variants were classified as deleterious if they were labelled as “deleterious”. For PolyPhen, variants were classified as deleterious if they were labelled as “probably damaging” or “damaging”. Our conservative Bonferroni adjusted significance level of the primary analyses for 119 genes and 6 gene sets was 0.05/(2 × 125) = 2.0E-4. Once considering our additional ten secondary tests, our Bonferroni adjusted significance level was 0.05/(12 × 125) = 3.3E-5. We repeated all primary and secondary tests using SKAT-O **(**Table 1 **;** Supplementary Table 4–9, 13).

### Functional studies of variants in ANGPTL6

To predict the impact of variants on the ANGPTL6 protein, we modelled their location using the crystal structure of a related protein fibrinogen in a complex with a peptide Gly-His-Pro-amide (described in Yang *et al*.^55^, PDB number: 1LWU, www.rcsb.org). The structural representations using ribbon-depicted models were generated using the Open-Source PyMOL Molecular Graphics System, Version 1.7.x Schrödinger, LLC (http://pymol.org). N-terminal triple Flag tag was added to human *ANGPTL6* cDNA cloned into a pEZ-M14 mammalian expression vector (Capital Bioscience). Mutations were introduced into this construct using QuikChange (Agilent technologies), and confirmed by Sanger sequencing. ANGPTL6 protein expression was studied by transient transfection in HEK293 cells. Media was collected 48 h – post transfection, the cell medium was centrifuged for 5 min (5,000 g at 4 °C) and the supernatants were collected. Cells were harvested prior to centrifugation for 15 min (15,000 g at 4 °C). Aliquots from the medium and cells were subjected to SDS-PAGE and immunoblot analysis. Primary antibodies (monoclonal Flag M2 antibody (Sigma) and a polyclonal antibody to Calnexin (Cell signalling)) were used at 1:1000, and secondary Horseradish peroxidase–conjugated anti-mouse or anti-rabbit IgG (Dako) antibody at 1:2000 dilution. After staining with ECL West Dura Substrate kit (Thermo Scientific), visualisation was performed on Chemidoc Digital Imager (Bio-Rad) (Supplementary Information). The relative protein secretion of ANGPTL6 for each variant was standardized to wild-type protein secretion. Experiments were performed in triplicate and analysed using an unpaired T-test with Welch’s correction.

### ANGPTL6 statistical follow-up analyses

We ran gene-based analyses using SKAT-O as described above on two subsets of ANGPTL6 variants (Supplementary Table 10): (1) the one variant that resulted in a complete loss of protein secretion (S284X; rs201622589), and (2) excluding S284X. For (1), we also used a Fisher’s Exact Test. We repeated the Fisher’s Exact Test of the S284X variant including additional population controls with pre-existing genotype data and replication samples (Online Methods, Sample Sets; Supplementary Tables 11 and 12).

### Power Analysis

We calculated the power to detect association to a gene region using the burden test with the *Power_Logistic_R* function and the haplotype dataset within the SKAT R-package^54^. We performed power calculations using 500 simulations on a random 2Kb sub region. The effect sizes of the causal variants are equal to log_10_(MAF) with a maximum effect size of 1.6 (MAF = 0.0001) and all have the same direction of effect. We limited the MAF threshold for causal variants to ≤ 0.01, 0.001, 0.0005 and varied the percentage of causal variants in the region to be between 10–90%. (More details in Supplementary Note 4).

## Electronic supplementary material

Supplementary Tables

Supplementary Information

## References

[1] El-Sayed Moustafa JS, Froguel P (2013). From obesity genetics to the future of personalized obesity therapy. Nat Rev Endocrinol.

[2] van der Klaauw AA, Farooqi IS (2015). The hunger genes: pathways to obesity. Cell.

[3] Wheeler E (2013). Genome-wide SNP and CNV analysis identifies common and low-frequency variants associated with severe early-onset obesity. Nat Genet.

[4] Consortium UK (2015). The UK10K project identifies rare variants in health and disease. Nature.

[5] Farooqi IS (2003). Clinical spectrum of obesity and mutations in the melanocortin 4 receptor gene. N Engl J Med.

[6] Lemos MC, Thakker RV (2015). GNAS mutations in Pseudohypoparathyroidism type 1a and related disorders. Hum Mutat.

[7] Aldred MA, Trembath RC (2000). Activating and inactivating mutations in the human GNAS1 gene. Hum Mutat.

[8] Warner DR, Weng G, Yu S, Matalon R, Weinstein LS (1998). A novel mutation in the switch 3 region of Gsalpha in a patient with Albright hereditary osteodystrophy impairs GDP binding and receptor activation. J Biol Chem.

[9] Bastida Eizaguirre M (2001). [Albright hereditary osteodystrophy: identification of a novel mutation in a family]. An Esp Pediatr.

[10] Chen M (2009). Central nervous system imprinting of the G protein G(s)alpha and its role in metabolic regulation. Cell Metab.

[11] Lee S, Wu MC, Lin X (2012). Optimal tests for rare variant effects in sequencing association studies. Biostatistics.

[12] Moutsianas L (2015). The power of gene-based rare variant methods to detect disease-associated variation and test hypotheses about complex disease. PLoS Genet.

[13] Oike Y (2005). Angiopoietin-related growth factor antagonizes obesity and insulin resistance. Nat Med.

[14] Kadomatsu T, Tabata M, Oike Y (2011). Angiopoietin-like proteins: emerging targets for treatment of obesity and related metabolic diseases. FEBS J.

[15] Purcell SM (2014). A polygenic burden of rare disruptive mutations in schizophrenia. Nature.

[16] Pinto D (2014). Convergence of genes and cellular pathways dysregulated in autism spectrum disorders. Am J Hum Genet.

[17] Krumm N (2015). Excess of rare, inherited truncating mutations in autism. Nat Genet.

[18] Farooqi IS (1999). Effects of recombinant leptin therapy in a child with congenital leptin deficiency. N Engl J Med.

[19] Kuhnen P (2016). Proopiomelanocortin Deficiency Treated with a Melanocortin-4 Receptor Agonist. N Engl J Med.

[20] Rasmussen SG (2011). Crystal structure of the beta2 adrenergic receptor-Gs protein complex. Nature.

[21] Tesmer JJ, Sunahara RK, Gilman AG, Sprang SR (1997). Crystal structure of the catalytic domains of adenylyl cyclase in a complex with Gsalpha.GTPgammaS. Science.

[22] Lynn, P. Sample design for Understanding Society. *Understanding Society Working Paper Series* **2009–01**(2009).

[23] Day N (1999). EPIC-Norfolk: study design and characteristics of the cohort. European Prospective Investigation of Cancer. Br J Cancer.

[24] Kamstrup PR, Tybjaerg-Hansen A, Steffensen R, Nordestgaard BG (2009). Genetically elevated lipoprotein(a) and increased risk of myocardial infarction. JAMA.

[25] Nordestgaard BG, Benn M, Schnohr P, Tybjaerg-Hansen A (2007). Nonfasting triglycerides and risk of myocardial infarction, ischemic heart disease, and death in men and women. JAMA.

[26] Varbo A (2013). Remnant cholesterol as a causal risk factor for ischemic heart disease. J Am Coll Cardiol.

[27] Danesh J (2007). EPIC-Heart: the cardiovascular component of a prospective study of nutritional, lifestyle and biological factors in 520,000 middle-aged participants from 10 European countries. Eur J Epidemiol.

[28] Sudlow C (2015). UK biobank: an open access resource for identifying the causes of a wide range of complex diseases of middle and old age. PLoS Med.

[29] Exome Aggregation Consortium (ExAC), Cambridge, MA *(URL:* http://exac.broadinstitute.org*)* ([September, 2015]).

[30] Hinney A (2007). Genome Wide Association (GWA) Study for Early Onset Extreme Obesity Supports the Role of Fat Mass and Obesity Associated Gene (FTO) Variants. PLoS ONE.

[31] Quante M (2012). The LIFE child study: a life course approach to disease and health. BMC Public Health.

[32] Korner A, Berndt J, Stumvoll M, Kiess W, Kovacs P (2007). TCF7L2 gene polymorphisms confer an increased risk for early impairment of glucose metabolism and increased height in obese children. J Clin Endocrinol Metab.

[33] Grozeva D (2014). De novo loss-of-function mutations in SETD5, encoding a methyltransferase in a 3p25 microdeletion syndrome critical region, cause intellectual disability. Am J Hum Genet.

[34] Li H (2009). The Sequence Alignment/Map format and SAMtools. Bioinformatics.

[35] Danecek P (2011). The variant call format and VCFtools. Bioinformatics.

[36] Genomes Project C (2010). A map of human genome variation from population-scale sequencing. Nature.

[37] Tennessen JA (2012). Evolution and functional impact of rare coding variation from deep sequencing of human exomes. Science.

[38] McLaren W (2010). Deriving the consequences of genomic variants with the Ensembl API and SNP Effect Predictor. Bioinformatics.

[39] Ng PC, Henikoff S (2003). SIFT: Predicting amino acid changes that affect protein function. Nucleic Acids Res.

[40] Ng PC, Henikoff S (2006). Predicting the effects of amino acid substitutions on protein function. Annu Rev Genomics Hum Genet.

[41] Sunyaev S, Ramensky V, Bork P (2000). Towards a structural basis of human non-synonymous single nucleotide polymorphisms. Trends Genet.

[42] Sunyaev S (2001). Prediction of deleterious human alleles. Hum Mol Genet.

[43] Gonzalez-Perez A, Lopez-Bigas N (2011). Improving the assessment of the outcome of nonsynonymous SNVs with a consensus deleteriousness score, Condel. Am J Hum Genet.

[44] Eilbeck K (2005). The Sequence Ontology: a tool for the unification of genome annotations. Genome Biol.

[45] Mungall CJ, Batchelor C, Eilbeck K (2011). Evolution of the Sequence Ontology terms and relationships. J Biomed Inform.

[46] Jun G (2012). Detecting and estimating contamination of human DNA samples in sequencing and array-based genotype data. Am J Hum Genet.

[47] Price AL (2006). Principal components analysis corrects for stratification in genome-wide association studies. Nat Genet.

[48] Wang C, Zhan X, Liang L, Abecasis GR, Lin X (2015). Improved ancestry estimation for both genotyping and sequencing data using projection procrustes analysis and genotype imputation. Am J Hum Genet.

[49] Purcell S (2007). PLINK: a tool set for whole-genome association and population-based linkage analyses. Am J Hum Genet.

[50] Online Mendelian Inheritance in Man, OMIM®. McKusick-Nathans Institute of Genetic Medicine, Johns Hopkins University (Baltimore, MD), (http://omim.org/) (February, 2013).

[51] The Mouse Genome Database (MGD), Mouse Genome Informatics. *The Jackson Laboratory, Bar Harbor, Maine*. http://www.informatics.jax.org (February, 2013).

[52] The Rat Genome Browser (RGD), Rat Genome Database Web Site. *Medical College of Wisconsin, Milwaukee, Wisconsin* http://rgd.mcw.edu/ (February, 2013).

[53] Wu MC (2011). Rare-variant association testing for sequencing data with the sequence kernel association test. Am J Hum Genet.

[54] Seunggeun Lee, with contributions from Larisa Miropolsky and Michael Wu. SKAT: SNP-Set(Sequence) Kernel Association Test.. *R package version 1.1.2*. http://CRAN.R-project.org/package=SKAT (2015).

[55] Yang Z (2002). Crystal structure of fragment D from lamprey fibrinogen complexed with the peptide Gly-His-Arg-Pro-amide. Biochemistry.

