# Supplementary material for: Rare Variant Analysis of Human and Rodent Obesity Genes in Individuals with Severe Childhood Obesity
Source: Sci Rep. 2017 Jun 29;7:4394. doi: 10.1038/s41598-017-03054-8 (PMC5491520; doi:10.1038/s41598-017-03054-8)
Supplement: Supplementary file 2 — Supplementary Information [file 41598_2017_3054_MOESM2_ESM.pdf]

Supplementary Information

Rare Variant Analysis of Human and Rodent Obesity Genes in Individuals with Severe Childhood Obesity

Audrey E. Hendricks[1,2*], Elena G. Bochukova[3*], Gaëlle Marenne[1], Julia M. Keogh[3], Neli Atanassova[3], Rebecca Bounds[3], Eleanor Wheeler[1], Vanisha Mistry[3], Elana Henning[3], Understanding Society Scientific Group[4,5], Antje Körner[7,8], Dawn Muddyman[1], Shane McCarthy[1], Anke Hinney[6], Johannes Hebebrand[6], Robert A. Scott[9], Claudia Langenberg[9], Nick J. Wareham[9], Praveen Surendran[10], Joanna MM Howson[10], Adam S. Butterworth[10,11], John Danesh[1,10,11], EPIC-CVD Consortium[12], Børge G Nordestgaard[13,14], Sune F Nielsen[13,14], Shoaib Afzal[13,14], Sofia Papadia[3], Sofie Ashford[3], Sumedha Garg[3], Glenn L. Millhauser[15], Rafael I. Palomino[15], Alexandra Kwasniewska[3], Ioanna Tachmazidou[1], Stephen O'Rahilly[3], Eleftheria Zeggini[1], UK10K Consortium[16], Inês Barroso[1,3], I. Sadaf Farooqi[3]

Affiliations:
[1]Wellcome Trust Sanger Institute, Cambridge, UK; [2]Department of Mathematical and Statistical Sciences, University of Colorado-Denver, Denver, CO 80204, USA; [3]University of Cambridge Metabolic Research Laboratories and NIHR Cambridge Biomedical Research Centre, Wellcome Trust-MRC Institute of Metabolic Science, Addenbrooke's Hospital, Cambridge, UK; [4]Institute for Social and Economic Research; [5]University of Warwick; [6]Department of Child and Adolescent Psychiatry, Psychotherapy, and Psychosomatics, University Hospital Essen, University of Duisburg-Essen, Essen, Germany; [7]Center for Pediatric Research, University Children's Hospital Leipzig, University of Leipzig, Germany; [8]IFB Adiposity Diseases, Medical Faculty, University of Leipzig, Germany; [9]MRC Epidemiology Unit, Institute of Metabolic Science, University of Cambridge School of Clinical Medicine, Cambridge, UK; [10]Cardiovascular Epidemiology Unit, Department of Public Health and Primary Care, University of Cambridge, Cambridge, CB1 8RN UK; [11]The National Institute for Health Research Blood and Transplant Unit (NIHR BTRU) in Donor Health and Genomics at the University of Cambridge; [13]Department of Clinical Biochemistry and The Copenhagen General Population Study, Herlev and Gentofte Hospital, Copenhagen University Hospital, Denmark; [14]Faculty of Health and Medical Sciences, University of Copenhagen, Denmark; [15]Department of Chemistry & Biochemistry, UC Santa Cruz, Santa Cruz, CA 95064, USA; [16]UK10K consortium.

Correspondence should be addressed to: I. Sadaf Farooqi (isf20@cam.ac.uk) and Inês Barroso (ib1@sanger.ac.uk). * These authors contributed equally.

Supplementary Figures

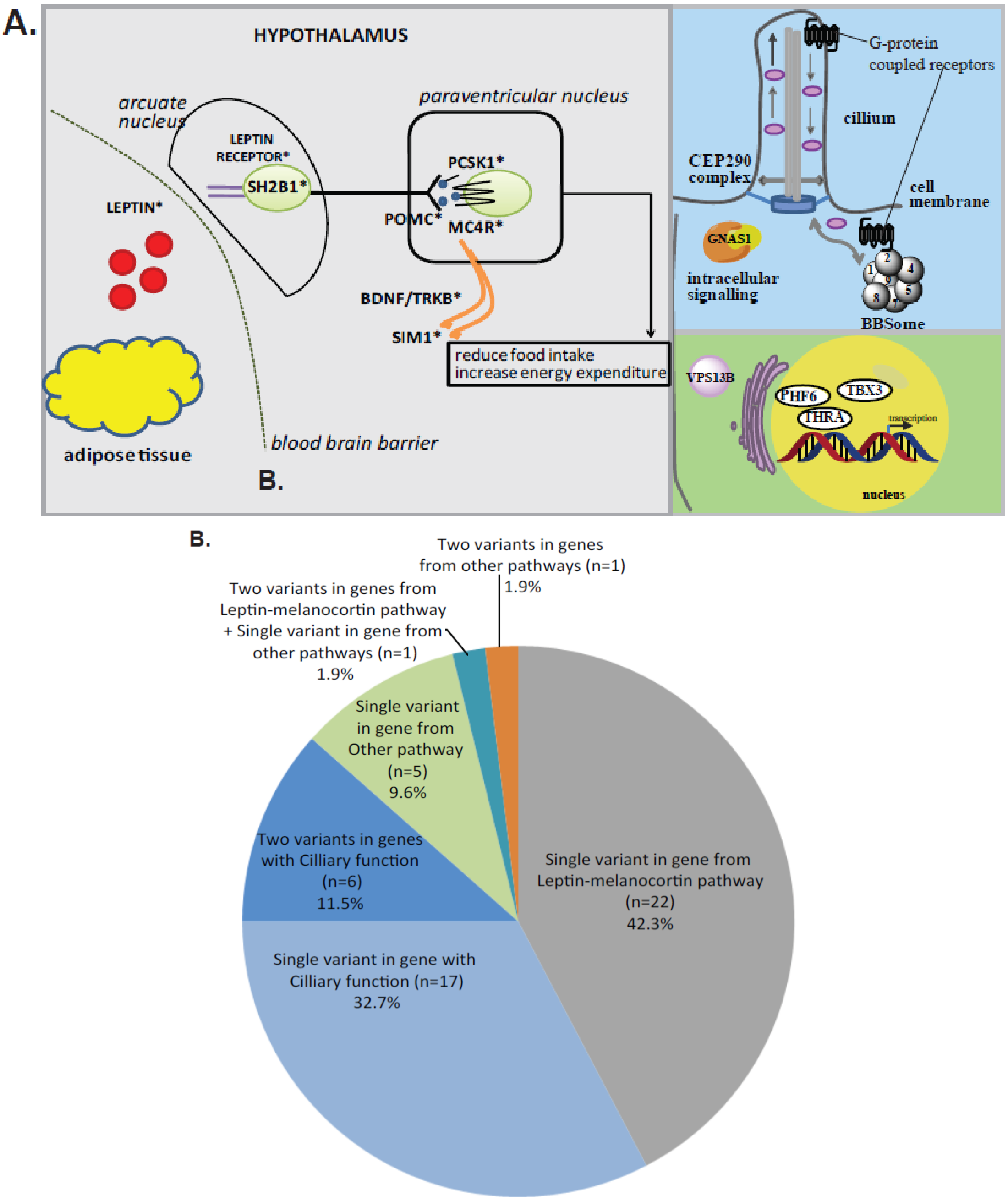

Supplementary Figure 1. A. Pathway representation grouping the known obesity genes by site and mode of action. Genes acting through the hypothalamic leptin-melanocortin pathway (grey), genes affecting ciliary function (blue) and other obesity syndrome genes (green). **B. Combination of genetic variants seen in individual patients (n=52) by pathway designation.**

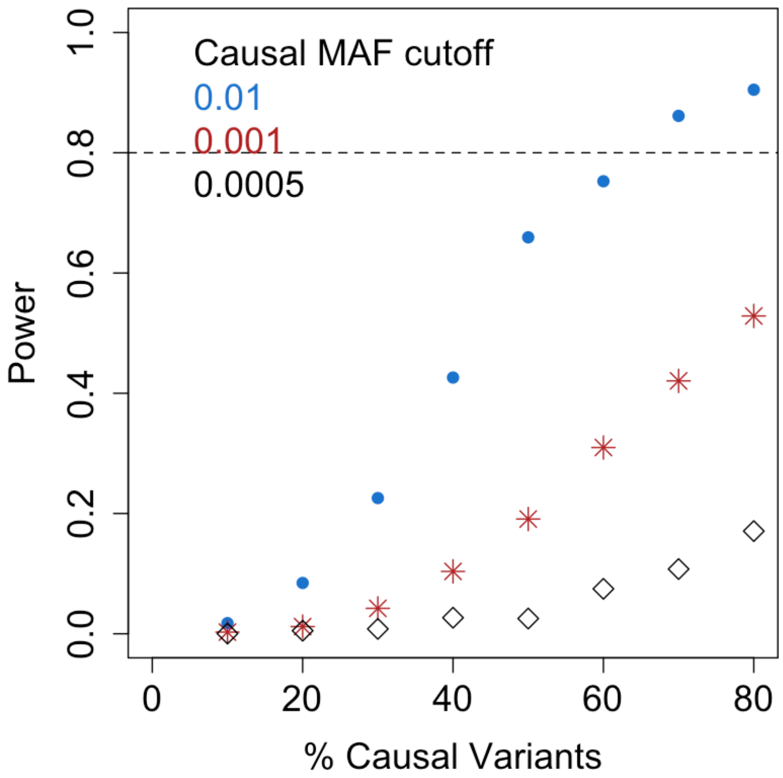

Supplementary Figure 2. **Gene-based power analysis.** Power to detect association to a gene region using a burden test across a variety of % causal variants as compared to the total number of variants (x-axis). We limited the MAF of causal variants to be below 0.01 (blue), 0.001 (red), or 0.0005 (black) (**Supplementary Note 4**).

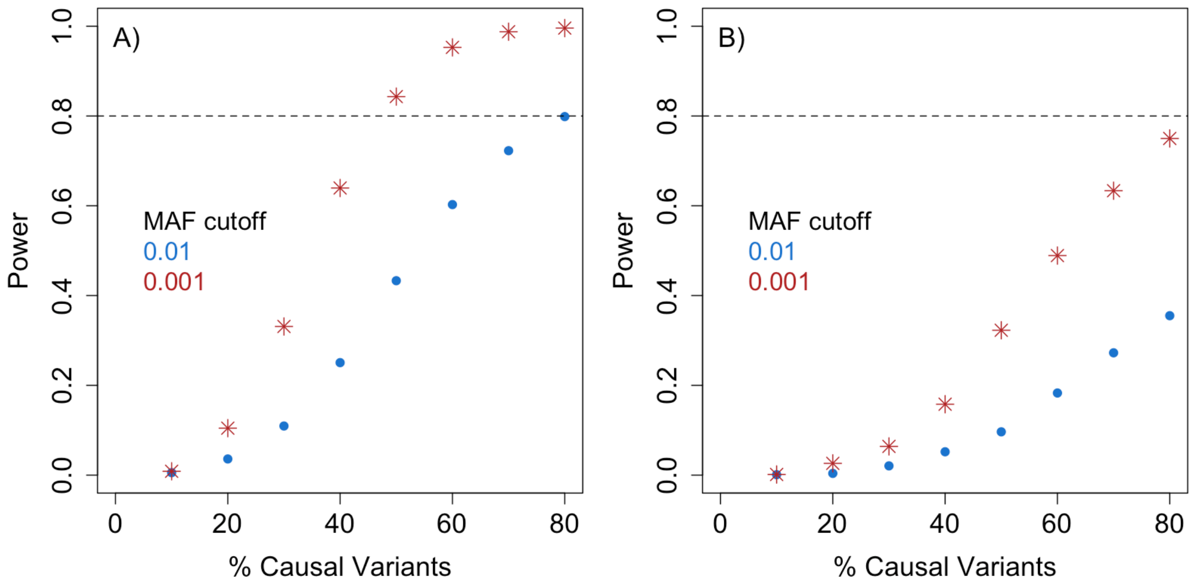

Supplementary Figure 3. **Effect of MAF threshold on gene-based power.** Power to detect association to a gene region using a burden test across a variety of % causal variants as compared to the total number of variants (x-axis). We limited the MAF of variants used in the burden test by limiting the MAF of the variants in the simulated haplotypes to be below

0.01 (blue) or 0.001 (red) (**Supplementary Note 4**). We further restricted the MAF of the causal variants to be below **(A)** 0.001 and **(B)** 0.0005.

SUPPLEMENTARY NOTES

1. Sample Set Descriptions

UK10K Sample Sets

We focus here on the SCOOP cohort as well as subsets within the neurodevelopmental and rare disease groups that were consented for use as controls (NEURO_MUIR, NEURO_IOP_COLLIER, NEURO_ABERDEEN, NEURO_ASD_GALLAGHER, NEURO_EDINBURGH, NEURO_GURLING,RARE_SIR, RARE_NEUROMUSCULAR, RARE_THYROID, RARE_HYPERCHOL). Also within the UK10K project, targeted sequencing (TS) was performed on a subset of the Genetics of Obesity Study (GOOS)[1] that was chosen to be mutually exclusive from the WES samples.

The thirteen UK10K WES sample sets that were not obese sample sets and were not included as controls in this study were used for variant filtering: NEURO_ASD_SKUSE, NEURO_ASD_TAMPERE, NEURO_ASD_BIONED, NEURO_ASD_MGAS, NEURO_FSZ, NEURO_FSZNK, NEURO_ASD_FI, NEURO_UKSCZ, NEURO_IMGSAC, RARE_COLOBOMA, RARE_CHD, RARE_CILIOPATHIES, and RARE_FIND.

Population Controls for ANGPTL6 Variant

For all population control samples except for ExAC, subjects overlapping with UK Biobank were removed.

The United Kingdom Household Longitudinal Study (UKHLS)[2] *(N = 9204)*

The UKHLS, also known as Understanding Society (https://www.understandingsociety.ac.uk) is a longitudinal panel survey of 40,000 UK households (England, Scotland, Wales, and Northern Ireland) representative of the UK population[2]. Participants are surveyed annually since 2009 and contribute information relating to their socioeconomic circumstances, attitudes, and behaviours via a computer assisted interview. The study includes phenotypical data for a representative sample of participants, for a wide range of social and economic indicators, as well as a biological sample collection encompassing biometric, physiological, biochemical, and haematological measurements and self-reported medical history and medication use. The UKHLS has been approved by the University of Essex Ethics Committee and informed consent was obtained from every participant.

In total, 10,484 samples were genotyped on the Illumina HumanCoreExome chip (v1.0) at the Wellcome Trust Sanger Institute. Genotype calling was performed using GenCall and zCall. We excluded samples with a call rate <98% and <99% for Gencall and zCall respectively, or that were heterozygosity outliers, had sex discrepancies, were duplicates or that were ethnic outliers. Variants were excluded with a call rate below 95% and 99% for GenCall and zCall respectively, with a Hardy-Weinberg equilibrium P < 10-4 or with a cluster separation score < 0.4. Prior to phasing we compared the variants to the 1000 Genomes Project and the UK10K haplotypes and we excluded any variant for which the alleles differed for the same variant at the same position. In addition variants were excluded if they were a duplicate, monomorphic, a singleton or known to have poor clustering after inspecting the intensity data. Samples were phased using SHAPEITv2 and imputed using IMPUTE v2. Unrelated samples were determined by performing identity by descent using only the autosomal directly genotyped variants with MAF≥1% and filtered so that only variants with a linkage-disequilibrium r-squared value <0.2 remained.

Fenland *(N = 8909)*

The Fenland Study is an ongoing, population-based cohort study (started in 2005) designed to investigate the association between genetic and lifestyle environmental factors and the risk of obesity, insulin sensitivity, hyperglycemia and related metabolic traits in men and women aged 30 to 55 years. Participants were recruited from General Practice sampling frames in the Fenland, Ely and Cambridge areas of the Cambridgeshire Primary Care Trust in the UK. Participants attended after an overnight fast for a detailed clinical examination, and blood samples were collected. Individuals were genotyped on the Affymetrix UK Biobank Axiom array and calling performed using Affymetrix Power Tools v1.16 following the Affymetrix best practices pipeline. The call rate for rs201622589 was 99.96%. http://www.mrc-epid.cam.ac.uk/ research/studies/fenland/

The European Prospective Investigation into Cancer (EPIC) Norfolk[3] *(N = 21014)*

The EPIC-Norfolk study is a cohort study investigating the relationship between diet and incident disease. Over 25,639 men and women aged between 45 and 74 were recruited in Norwich and the surrounding area. Individuals were characterized for cardiovascular and metabolic phenotypes. Individuals were genotyped on the Affymetrix UK Biobank Axiom array and calling performed using Affymetrix Power Tools v1.16 following the Affymetrix best practices pipeline. The call rate for rs201622589 was 100% in EPIC Norfolk. http://www.epic-norfolk.org.uk/

Copenhagen Ischemic Heart Disease Study (CIHDS)[4-6] *(N = 2450)*

This study involves participants with myocardial infarction and other major acute coronary syndromes. The participants were recruited from Copenhagen University Hospital during the period from 1991 to 2009. In addition to a diagnosis of acute coronary syndrome, these cases also had stenosis or atherosclerosis on coronary angiography and/or positive results on exercise electrocardiography. Cases were classified by World Health Organization International Classification of Diseases-Eighth Revision, codes 410 to 414; International Classification of Diseases-Tenth Revision, codes I20 to I25, and through review of all hospital admissions and diagnoses entered in the national Danish Patient Registry and all causes of death entered in the national Danish Causes of Death Registry, as previously described.

Copenhagen General Population Study (CGPS)[4-6] *(N=11803)*

The CGPS is a population-based prospective study initiated in 2003 with ongoing enrolment. Participants were selected on the basis of the national Danish Civil Registration System to reflect the adult Danish population age 20 to ≥80 years. Data were obtained from a questionnaire, a physical examination, and blood samples including DNA extraction. Follow-up was 100% complete; that is, no participant was lost to follow-up.

Copenhagen City Heart Study (CCHS)[4-6] *(N = 8080)*

CCHS is a population-based prospective study initiated in 1976 with follow-up examinations from 1981 to 1983, 1991 to 1994, and 2001 to 2003. Selection of individuals for the CCHS was based on the same criteria as for the CGPS. Information on diagnosis of CHD (defined as WHO ICD 8 410 to 414 and WHO-ICD 10 I20 to I25) was collected and verified from 1976 until 2010 by reviewing all hospital admissions and diagnoses entered in the national Danish Patient Registry, and by reviewing all causes of death entered in the national Danish Causes of Death Registry. Again, follow-up was 100% complete for both non-fatal coronary outcomes and mortality.

European Prospective Investigation into Cancer and Nutrition-CVD (EPIC-CVD)[7] *(N=19584)*

EPIC is a multi-centre prospective cohort study of 519,978 participants (366,521 women and 153,457 men, mostly aged 35–70 years) recruited between 1992 and 2000 in 23 centres located in 10 European countries. Participants were invited mainly from population-based registers (Denmark, Germany, certain Italian centres, the Netherlands, Norway, Sweden, UK).

Other sampling frameworks included: blood donors (Spain and Turin and Ragusa in Italy); screening clinic attendees (Florence in Italy and Utrecht in the Netherlands); people in health insurance programmes (France); and health conscious individuals (Oxford, UK). About 97% of the participants were of white European ancestry. Prevalent CHD was ascertained through self-reported history of MI or angina, or registry-ascertained CHD event prior to baseline. EPIC-CVD employs a nested case-cohort design, analogous to the EPIC-InterAct study for type-2 diabetes which established a common set of referents through selection of a random sample of the entire cohort ("subcohort"). Incident CHD cases have been defined as fatal and non-fatal MI and other major acute coronary events, according to ICD-10 codes I20-I25. All centres have recorded cause-specific mortality through mortality registries and/or active follow-up, and have ascertained and validated incident fatal and non-fatal CHD through a combination of methods (eg, morbidity registers, general practice records, MONICA registries, self-report, clinical records). For the overall analysis, samples within EPIC-CVD that overlapped with EPIC Norfolk were removed.

Genotyping and QC for CCHS, CGPS, CIHDS, and EPIC-CVD

Samples were genotyped in batches at the Herlev Hospital in Copenhagen (CCHS, CGPS and CIHDS) or Cambridge Genomic Services (EPIC-CVD) on customised versions of the Illumina Human Exome v1.1 SNP array. Genotype calling was performed centrally for all batches at the University of Cambridge using optiCall (0.7.0), followed by zCall for variants with minor allele frequency (MAF) <5%.

Samples with extreme intensity values, and outlying plates or arrays were removed prior to all genotype calling. Samples with call rates more than 3 standard deviations below the mean were removed prior to post-processing optiCall calls with zCall. Within each batch, variants were removed if variant call rate < 0.97; HWE P $<1x10^{-6}$ for common variants or HWE P $<1x10^{-15}$ for variants with MAF<0.05. Variants within each genotyping batch were aligned to human genome reference sequence plus strand and the standardized files were then used for sample QC. Samples were excluded from each batch/study if sample heterozygosity > ±3 standard deviations from the mean heterogeneity or sample call rate >3 standard deviations from the mean call rate. Duplicates within each batch and ancestral outliers identified by PCA were removed. Samples and variants that failed QC were removed from individual batches. Where studies were analyzed in multiple batches, the batches were combined and any variants out of HWE across the study as a whole were also removed.

UK Biobank[8] *(N=139183)*

500,000 participants aged 40-69 years were recruited between 2006 and 2010 in 22 assessment centres throughout the UK. The assessment visit included electronic signed consent, a self-completed touch-screen questionnaire, brief computer-assisted interview, physical and functional measures, and collection of biological samples and genetic data (http://www.ukbiobank.ac.uk/wp-content/uploads/2011/11/UK-Biobank-Protocol.pdf).

BMI was calculated (kg/m^2) using measured height and weight. Weight (kg) was measured using the Tanita BC-418 MA body composition analyser (accurate to within 0.1kg) after removal of heavy clothing and shoes. Standing height (cm) was measured without shoes using a Seca 202 height measure.

All the analyses were carried out in a set of unrelated, Europeans. Subjects with high heterozygosity, low call rate, and pregnant women were excluded from the analysis. SNP genotypes were called by Affymetrix and SNPs that failed Affymetrix batch-specific QC thresholds were set to missing in all subjects from that batch. Additional SNP QC steps were carried out by the UK Biobank team, in which SNPs at certain batch/plates were set to missing

if the genotype distributions were significantly different from other batches/plates (p-value < 10^{-12}), or there were significant deviations of genotype frequencies from those expected under Hardy-Weinberg equilibrium (p-value < 10^{-12}). Imputation was carried out using the 1000 Genomes phase 3 and UK10K combined reference panel.

Exome Aggregation Consortium (ExAC)[9] *(N=33,360)*

We accessed the ExAC browser (http://exac.broadinstitute.org/) on September 22, 2015 searching for the variant "rs201622589". We recorded the allele count, allele number, and number of homozygotes for the Non-Finnish European population (39; 66720; 0 respectively; N=33,360).

Obesity case-control replication studies

For the Hinney study, genotyping of the variant was performed by Taqman assay C_190494905_10 in 487 extremely obese children and adolescents ("cases") recruited in hospitals specialized for the inpatient treatment of extreme obesity (mean BMI Z score: 4.63±2.27; age in years: 14.38±3.74) and in 442 healthy lean individuals ("controls") ascertained at the University of Marburg (mean BMI Z score: -1.38±0.35; age in years: 26.07±5.79). More information about this sample can be found in Hinney et al[10].

For the Korner study, the variant was genotyped on 949 obese children (BMI Z score > 1.88, mean 2.60±0.52; age in years 11.95±4.36), 271 overweight children (1.88 ≥ BMI Z score > 1.23, mean 1.60±0.18; age in years 11.58±2.98), and 1513 lean children (BMI Z score ≤ 1.23, mean -0.18±0.83; age in years 11.58±3.36) from the Leipzig Childhood Obesity Cohort[11] and LIFE Child cohorts [12]. Genotyping was performed using Illumina platform applying Infinium® Human Exome-12v1.2 bead chips. All guardians approved the study and gave informed consent. Studies are registered under NCT01605123 and NCT02550236 respectively. All subjects underwent clinical exam including anthropometric measurements and metabolic assessment by standard oral glucose tolerance test. Children with known syndromal forms of obesity (e.g. Prader-Willi-Syndrome) were excluded.

2. Sequencing

Prior to targeted sequencing, TS samples were whole-genome amplified (GenomiPhi V2 DNA Amplification Kit; GE Healthcare, Little Chalfont, Buckinghamshire, United Kingdom) with the use of 1 µl of 10 ng/µl template DNA prior to pull-down.

Exome and targeted sequencing was performed with DNA (1-3µg) sheared to 100-400 bp using a Covaris E210 or LE220 (Covaris, Woburn, MA, USA). Sheared DNA was subjected to Illumina paired-end DNA library preparation and enriched for target sequences (Agilent Technologies; Human All Exon 50 Mb - ELID S02972011 for WES, and a custom-based targeted Agilent SureSelect Human All Exon V5 - ELID S04380110 for TS) according to manufacturer's recommendations (Agilent Technologies; SureSelectXT Automated Target Enrichment for Illumina Paired-End Multiplexed Sequencing for WES, and HaloPlex Target Enrichment Kit for TS).

For WES, the bait regions covered 49.4 Mb on the autosomes using 204,609 intervals and 2.1 Mb on the sex chromosomes using 8,338 intervals. For TS, the bait regions covered 3.8 Mb on the autosomes using 16,277 intervals and 159.3 kb on the X chromosome using 691 intervals. Here, we focused on a subset of 119 from both the TS and WES studies. Enriched libraries were sequenced using the HiSeq platform (Illumina) as paired-end 75 base reads according to manufacturer's protocol. Details about alignment and BAM processing are available in the 2015 UK10K paper [13]. All alignment, processing, and variant calling was done using GRCh37.

Variant Calling

A BCF file was created and the genotype likelihoods were calculated for each site using the following command.

samtools mpileup -EDVS -C50 -pm3 -F0.2 -d2000 -L500 -P ILLUMINA -g -l bedfile.bed

Then variants (SNPs and Indels) were called using BCFtools using the following command.

bcftools view -vcgNm0.99

Variant Quality Control

Variants were filtered both at the site and genotype level. Sites were removed from further analysis using the filters show below in *vcf-annotate*[14].

StrandBias,Description="Min P-value for strand bias (INFO/PV4) [0.0001]
EndDistBias,Description="Min P-value for end distance bias (INFO/PV4) [0.0001]
MaxDP,Description="Maximum read depth (INFO/DP or INFO/DP4) [10000000]
BaseQualBias,Description="Min P-value for baseQ bias (INFO/PV4) [0]
MinMQ,Description="Minimum RMS mapping quality for SNPs (INFO/MQ) [10]
MinAB,Description="Minimum number of alternate bases (INFO/DP4) [2]
Qual,Description="Minimum value of the QUAL field [20]
VDB,Description="Minimum Variant Distance Bias (INFO/VDB) [0]
GapWin,Description="Window size for filtering adjacent gaps [3]
MapQualBias,Description="Min P-value for mapQ bias (INFO/PV4) [0]
SnpGap,Description="SNP within INT bp around a gap to be filtered [5]
RefN,Description="Reference base is N []
MinDP,Description="Minimum read depth (INFO/DP or INFO/DP4) [8000]

Calling across all samples results in genotype calls at all sites for all samples even when there is little evidence for a site for a particular sample. To mitigate this we set the genotypes to missing at the individual level when depth or genotype quality for that particular genotype call fell outside given ranges (i.e. Indel depth < 4 or Indel depth > 2000; Indel genotype quality < 60; SNP genotype quality < 20).

3. Sample quality control

Identifying non-European samples

To identify non-European samples, we calculated principal components (PCs) from the 1000Genomes Phase I integrated call set[15] using either EIGENSTRAT v4.2[16] or LASER 2.0[17] for the WES and TS samples respectively.

For the WES samples, we used a set of biallelic SNVs determined to be both common (MAF > 5%) and high-quality in both 1000Genomes and the WES samples. 1000Genomes high-quality variants were defined as being in region P of the strict accessibility mask defined by 1000Genomes[15], genotype imputation quality RSQ>0.9, and HWE p-value < $1x10^{-6}$. WES high-quality variants were defined as passing all variant quality thresholds, and having a genotype call rate > 95%. The remaining SNPs were LD-pruned in PLINK using the command –indep 50 5 2 leaving 17,850 SNPs to calculate the PCs in EIGENSTRAT [16].

For the 2,676 TS samples remaining after sample quality control, we used a subset of biallelic, common (MAF > 5%), and high-quality SNVs used for WES samples. We randomly selected 1,500,000 of the off-target SNVs (>100bp of the start or the end of any probe). We then LD-pruned these SNVs in PLINK using the command -- *indep-pairwise 50 5 0.5* leaving 418,163 SNVs to calculate the PCs in LASER [17].

For both the WES and TS samples, we projected the samples back onto the PC space created from 1000Genomes. A circle was created centred at the mean of PC1 and the mean of PC2 for

the 1000Genomes European samples (i.e. CEU, TSI, IBS, and TSI) with a radius 1.5 times the maximum distance of these 1000Genomes European samples to the centre. Samples outside of this circle were defined as non-European samples. Of the 837 TS samples classified as non-European due to genetic ancestry, the majority also had non-European reported ancestry (N = 568).

Identifying relatedness for TS samples

To identify genetic relationships within the TS sample set and between the TS and WES sample sets, a subset of high quality, common SNPs (GCR > 95%, HWE p-value > 1e-4, biallelic, and MAF > 1%) within the TS regions were used. This resulted in only 4,370 SNPs; a much smaller number of SNPs than is often used to estimate IBD. To ensure that the IBD estimation from the TS+WES samples was robust to using a small number of SNPs, we calibrated the IBD threshold for the TS+WES samples to that from the WES samples only. For over 1.7 million WES subject pairs, IBD estimates were calculated twice using either the smaller SNP set from the TS+WES samples or the larger SNP set from the WES only samples. The vast majority (~99.9%) of these sample pairs were unrelated.

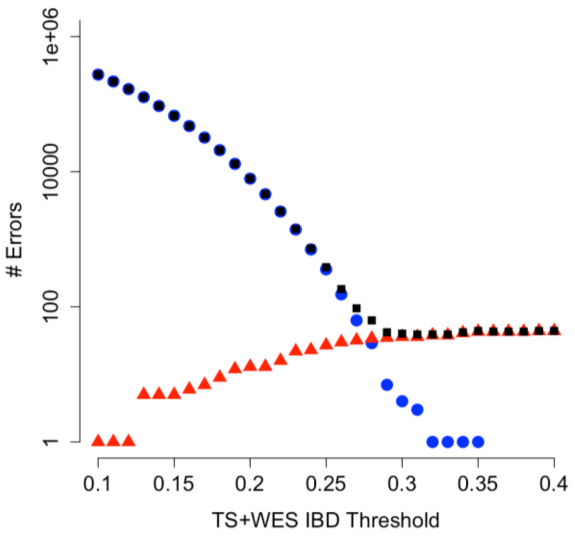

Using the larger WES SNP set and estimated IBD threshold of ≥ 0.125 as the "gold standard" to classify related pairs, we calculated the number of false positive relationships (Figure; blue) or missed relationships (Figure; red) over various IBD thresholds for the smaller TS+WES SNP set. As seen in the Figure, the number of total errors (Figure; black) decreases as the IBD threshold for the TS+WES sample set increases leveling out at an IBD threshold of approximately 0.3. Thus, subjects within the TS sample set or between the TS and WES sample sets were classified as related if the estimated IBD ≥ 0.3.

4. Power Analysis

For these simulations we used the haplotype dataset contained within the SKAT package, which has 10,000 haplotypes of a 200Kb region simulated using a coalescent model assuming European ancestry. The region contains 3,845 single nucleotide variants of which the majority are rare (85%, 73%, 62% have a MAF ≤ 0.01, 0.001, 0.0005 respectively). First, we calculated the power of the burden test across various causal variant MAF thresholds (0.01, 0.001, 0.0005) and percent of causal variants in the region (10-90%). Then, we sought to determine whether the MAF threshold used to include variants in the gene-region test affected the power of the test when the causal variants are all very rare. Although highly flexible in other ways, this power function does not give the ability to provide a MAF threshold on which variants are included in the test. Thus, we limited the variants that were included in the test by limiting the original set of haplotypes from which the simulations were sampled to only contain variants below a certain MAF threshold (either 0.01 or 0.001). We equate this to using the MAF from publically available large datasets (such as 1000Genomes, UK10K, or NHLBI EVS) to filter to rare variants. We performed power calculations using 500 simulations for which a random 2Kb sub region was chosen each time usually containing ~20 variants for MAF ≤ 0.001. The effect sizes of the causal variants are equal to $\log_{10}$(MAF) with a maximum effect size of 1.6 (MAF = 0.0001).

References

1. Bochukova, E.G. *et al.* Large, rare chromosomal deletions associated with severe early-onset obesity. *Nature* **463**, 666-70 (2010).
2. Lynn, P. Sample design for Understanding Society. *Understanding Society Working Paper Series* **2009-01**(2009).
3. Day, N. *et al.* EPIC-Norfolk: study design and characteristics of the cohort. European Prospective Investigation of Cancer. *Br J Cancer* **80 Suppl 1**, 95-103 (1999).
4. Kamstrup, P.R., Tybjaerg-Hansen, A., Steffensen, R. & Nordestgaard, B.G. Genetically elevated lipoprotein(a) and increased risk of myocardial infarction. *JAMA* **301**, 2331-9 (2009).
5. Nordestgaard, B.G., Benn, M., Schnohr, P. & Tybjaerg-Hansen, A. Nonfasting triglycerides and risk of myocardial infarction, ischemic heart disease, and death in men and women. *JAMA* **298**, 299-308 (2007).
6. Varbo, A. *et al.* Remnant cholesterol as a causal risk factor for ischemic heart disease. *J Am Coll Cardiol* **61**, 427-36 (2013).
7. Danesh, J. *et al.* EPIC-Heart: the cardiovascular component of a prospective study of nutritional, lifestyle and biological factors in 520,000 middle-aged participants from 10 European countries. *Eur J Epidemiol* **22**, 129-41 (2007).
8. Sudlow, C. *et al.* UK biobank: an open access resource for identifying the causes of a wide range of complex diseases of middle and old age. *PLoS Med* **12**, e1001779 (2015).
9. Exome Aggregation Consortium (ExAC), Cambridge, MA *(URL: http://exac.broadinstitute.org)* ([September, 2015]).
10. Hinney, A. *et al.* Genome wide association (GWA) study for early onset extreme obesity supports the role of fat mass and obesity associated gene (FTO) variants. *PLoS One* **2**, e1361 (2007).
11. Korner, A., Berndt, J., Stumvoll, M., Kiess, W. & Kovacs, P. TCF7L2 gene polymorphisms confer an increased risk for early impairment of glucose metabolism and increased height in obese children. *J Clin Endocrinol Metab* **92**, 1956-60 (2007).
12. Quante, M. *et al.* The LIFE child study: a life course approach to disease and health. *BMC Public Health* **12**, 1021 (2012).
13. Consortium, U.K. *et al.* The UK10K project identifies rare variants in health and disease. *Nature* **526**, 82-90 (2015).
14. Danecek, P. *et al.* The variant call format and VCFtools. *Bioinformatics* **27**, 2156-8 (2011).
15. Genomes Project, C. *et al.* A map of human genome variation from population-scale sequencing. *Nature* **467**, 1061-73 (2010).
16. Price, A.L. *et al.* Principal components analysis corrects for stratification in genome-wide association studies. *Nat Genet* **38**, 904-9 (2006).
17. Wang, C., Zhan, X., Liang, L., Abecasis, G.R. & Lin, X. Improved ancestry estimation for both genotyping and sequencing data using projection procrustes analysis and genotype imputation. *Am J Hum Genet* **96**, 926-37 (2015).
